# Understanding the Structural-Dependent Photocatalytic Antibacterial Activity: a Case Study of Ag Modified BiVO_4_

**DOI:** 10.1186/s11671-020-03380-3

**Published:** 2020-07-22

**Authors:** Hailin Guan, Yuefeng Tian, Alideertu Dong, Yiguo Su

**Affiliations:** College of Chemistry and Chemical Engineering, Inner Mongolia University, Hohhot, 010021 Inner Mongolia People’s Republic of China

**Keywords:** Ag/BiVO_4_, Photocatalysis, Structural dependent, *E. coli* inactivation, DFT calculation

## Abstract

In this work, Ag/BiVO_4_ heterostructural photocatalysts were developed in order to reveal exceptional structural-dependent photoinduced charge migration kinetics as well as the underlying photocatalytic antibacterial dynamic process. The structure-dependent interface of BiVO_4_ and Ag nanoparticles was successfully constructed to improve the photoinduced interface charge transfer efficiency and interface correlation. DFT calculation indicated that a net charge of about 0.33 e between Ag and tz-BiVO_4_ was achieved by extraordinary interface charge transfer, being far larger than that between Ag and ms-BiVO_4_. Larger net charge has consequences on mobility of charge carriers of tz-BiVO_4_ that can raise the migration and separation of charge carriers for Ag/tz-BiVO_4_ heterojunction. Fine interfacial contact between Ag and tz-BiVO_4_ led to the optimized photocatalytic performance toward *E. coli* inactivation, being predominately higher than that of tz-BiVO_4_, ms-BiVO_4_, and Ag/ms-BiVO_4_ catalysts. Besides photocatalytic activity, the thermocatalytic inactivation activity of Ag/tz-BiVO_4_ also exhibited a factor of about 7.2 and 3.1 times higher than that of tz-BiVO_4_ and Ag/ms-BiVO_4_. Trapping and EPR measurements suggested that the structural-dependent photocatalytic activity of Ag/BiVO_4_ mainly originated from the pronounced variation of the capability to produce H_2_O_2_ active species, where the capability of generating H_2_O_2_ over Ag/tz-BiVO_4_ is highly accelerated. Moreover, it cannot be ignored that this study provides an ideal candidate for many aspects, such as environmental and water pollution caused by pathogenic microorganisms and disinfection of medical materials, food packaging, household materials, and public places, etc.

## Background

Utilization of solar light and semiconductors for photocatalytic purpose is still a research hotspot, which is found wide applications in energy conversion and environmental remediation [1, 2]. Nano-heterojunctions have also received attention that has to be taken seriously, because they can be applied to various aspects and have achieved extraordinary results in many potential applications [3–8]. Furthermore, due to its advantages of high efficiency, environmentally friendly, and renewable energy, photocatalytic antibacterial technology has an irreplaceable role in environmental governance and sterilization [9, 10]. Recently, BiVO_4_ emerges an excellent candidate because of its tunable crystal structure and appropriate electronic structure [11, 12]. However, despite the favorable structural characteristics of BiVO_4_, inefficient charge carrier transmission and short length of carrier diffusion are hindrance of its application in practice. From solid state physics viewpoint, the photocatalytic performance is thought to be predominately regulated by the distortion of microstructure. The tetragonal zircon (tz-) BiVO_4_ synthesized by ethylene-glycol colloidal path controllable at room temperature induces strongly improved photocatalytic activity over monoclinic scheelite (ms-) BiVO_4_, yet the underlying mechanism remains ambiguous [13]. Therefore, the modification of BiVO_4_ is not limited to improving the photocatalytic activity; it is also absolutely necessary to explain the kinetics of photoinduced charge transfer from the microstructure.

Frequently, localizations of excess charge carriers as the so-called localized polarons accompanied by microstructural and interfacial regulation have intensely emotional impact on charge carrier mobility of oxide semiconductors. Localized polarons in BiVO_4_ either inhibit the kinetics of charge mobility or influence the photocatalytic activity at surfaces [14, 15]. The reconstruction of surface or interface structures can drive the variation of polaron extension that affects the mobility of charge carriers as well as the photocatalytic performance. Noble metals, like Ag, Au, and et al., can act as photosensitizers to absorb visible light and regulate the generation of charge carrier through direct electron transfer or dipole-dipole coupling connection [16, 17]. The junction of noble metal with semiconductor to establish interfacial charge transfer provides an efficient approach to influence the polaron extension as well as the mobility of charge carriers that can raise the migration and separation of charge carriers. For instance, Au nanosphere decorated Mo:BiVO_4_ photoanode displays increased photocurrent intensity of about 2.2 times in comparison to Mo:BiVO_4_ [18]. Recent investigations on Ag/BiVO_4_ nanostructures demonstrate highly improved photocatalytic performance toward water oxidation, organic dye degradation, and so forth [19, 20]. Most reports very often merely concentrated on fine characterization of photocatalytic response but did not focus on the microstructural analyses which profoundly governed the native photophysical and photochemical performance of semiconductors. Considering to uncovering the structural-dependent native properties, experimental identification of phase structure as well as surface/interface feature of Ag/BiVO_4_ nanostructure is essential and advantageous to regulate the native properties and provides some hints to various structural-linked semiconductors.

Herein, this work means to deliver a proof by rationally controlling the phase structure of BiVO_4_ and assembling Ag nanoparticles for photocatalytic antibacterial purpose in order to reveal structural-dependent photoinduced charge migration as well as the underlying photocatalytic antibacterial dynamic process.

## Methods/Experimental

### Chemicals

Bismuth nitrate (Bi(NO_3_)_3_•5H_2_O) (purity 99%), silver nitrate (AgNO_3_) (purity 99.8%), and absolute ethanol (purity 99.7%) were obtained from Wind ship in Tianjin chemical reagent Co. Ltd (Tianjin, China). Ammonium metavanadate (NH_4_VO_3_) (purity 99.9%) was purchased from Adamas Reagent Co. Ltd (Shanghai, China). Distilled water was also needed. All reagents were used without further purification.

### Synthesis of BiVO_4_ and Ag-Loaded BiVO_4_

#### Synthesis of BiVO_4_

BiVO_4_ samples were prepared by hydrothermal method. One millimole of Bi(NO_3_)_3_ was added into 20 mL of distilled water under mild stirring, and a white suspension was formed for 30 min. One millimole of NH_4_VO_3_ was added into 40 mL of distilled water to form a white suspension with stirring for 30 min. Then, NH_4_VO_3_ suspension was dropwised into the Bi(NO_3_)_3_ solution to form an orange suspension. The pH value of the orange suspension is 0.59. Sodium hydroxide solution was adopted to adjust the pH value from 0 to 12 for the above suspension. And the suspension was loaded into a 100 Teflon-lined autoclave. The autoclave was sealed and heated in an oven at 180 °C for 12 h. The pH value of the suspension after the reaction was maintained. The autoclave was then naturally cooled to room temperature, where the obtained yellow powder was collected and washed with distilled water and ethanol several times to remove ions and possible remnants until the pH value near neutral and vacuum-dried for further characterization.

#### Synthesis of Ag-Loaded BiVO_4_

A set of five identical solutions was prepared, each of which contains 1 g BiVO_4_ mixed in 40 mL of ethanol and sonicated for 10 min. Another set of solutions containing an appropriate amount of AgNO_3_ was obtained. Then, AgNO_3_ aqueous solution was carefully dropped into BiVO_4_ solution and kept in the dark for 1 h with constant stirring. After that, the mixture of AgNO_3_ and BiVO_4_ was exposed to UV light for 2 h with stirring so as to Ag nanoparticles loaded BiVO_4_ samples. The samples were then dried overnight at 60 °C. The initial Ag loading content was fixed to be 1 wt%, 3 wt%, 5 wt%, 7 wt%, and 10 wt%.

### Bacteria Preparation

The lyophilized powder was dissolved, and 1 mL of the bacterial suspension was adhered to the solid culture plate with a hot-sterilized toothpick. The inoculated solid culture plate was inverted and placed in a 37 °C incubator for 12 h. Then, the selection of the single colony and the expansion of the culture are carried out. The final cell density was adjusted to about 1 × 10^7^–1 × 10^9^ colony forming unit (CFU) mL^−1^.

### Photocatalytic Bacterial Inactivation

The VLD photocatalytic inactivation of *Escherichia coli* (*E. coli* ATCC 8099, Gram-negative bacteria) and *Staphylococcus aureus* (*S. aureus* ATCC 25923, Gram-positive bacteria) by Ag/tz-BiVO_4_ was conducted under fluorescent tubes (PCX50C Discover) irradiation. A suspension (40 mL) containing the bacterial cells and the photocatalyst (40 mg). Then, the solution was turned on to start the photocatalytic inactivation experiments. At different time intervals, aliquots of the samples were collected and serially diluted with sterilized aqueous solution. Then, 1 mL of the diluted samples was immediately spread on Nutrient Agar plates and incubated at 37 °C for 12 h to determine the number of survival cells. *S. aureus* cultured at 54 °C for 24 h. For the comparison, light control (bacterial cells and light without photocatalyst) and dark control (photocatalyst and bacterial cells without light) were also conducted in the study.

The photocatalytic degradation performance of the prepared samples was evaluated by photodegradation of the MB (Methylene Blue) dye solution (5 mg/L, 30 mL) under visible light irradiation. A 300 W Xenon lamp equipped with a 420-nm cutoff filter was used as the light source. In the photodegradation experiment, 15 mg of photocatalysts was dispersed in 30 mL of the MB dye solution. To ensure the balance of adsorption and desorption, the quartz tube containing the solution was kept in the dark for 1 h before irradiation. At specific time intervals, 4 mL of the suspension was collected and analyzed by UV-visible diffuse reflectance spectrometer. The absorption peaks at 672 nm were employed to determine the concentration of the residual MB solution.

To identify the dominant reactive species accounting for the photocatalytic bacterial inactivation, specific compounds (i.e., respective scavengers) at predetermined optimized concentration were individually added into reaction solution with identical conditions mentioned above. All the above experiments were repeated in triplicates. At the same time, the capture experiment of photocatalytic degradation of MB solution was also performed.

### Preparation Procedure for SEM Observation of Bacteria

The mixtures of photocatalyst *E. coli* before and after the inactivation were firstly sampled and centrifuged and wash the bacteria solution twice with PBS (phosphate buffer saline). After this, the harvested cells were prefixed in 2.5% glutaraldehyde for 12 h. After washed with 0.1 M PBS, the specimens were dehydrated in a graded series of ethanol (20% for once, 50% for once, 80% for one time, 100% for one time) each for 10 min and then wash the sides with t-butanol. Finally, drop it onto a clean silicon wafer for SEM observation.

### Morphology, Structure, and Optical Properties Characterizations

Phase purity of all samples was characterized by X-ray diffraction (XRD) on Rigaku DMAX2500 X-ray diffractometer using a copper target (*λ* = 0.15406 nm). The scanning speed was 1° per minute, the scanning step was 0.05°, and the scanning range was set to 5–80°. Morphology of the samples was determined using scanning electron microscopy (SEM) on S4800 apparatus working at 10 kV and transmission electron microscopy (TEM) on a DHG-9240B FEI apparatus with an acceleration voltage of 200 kV. Appropriate amount of catalyst to be tested was dispersed it in absolute ethanol by ultrasonic dispersion. In the SEM test, the dispersed sample was dropped on a clean silicon wafer, and in the TEM test, it was dropped on a copper mesh supported by a carbon film. X-ray photoelectron spectroscopy (XPS) measurement were performed on a Thermo ESCALAB 250 with Al Ka (1486.6 eV) line at 150 W. To compensate for surface charges effects, the binding energies were calibrated using the C 1 s peak at 284.60 eV as the reference; the casaXPS program was used to realize the quantification of the elements. UV-visible diffuse spectra of the samples were measured using Lambda 750 s UV/vis spectrometer. Barium sulfate was chosen as the reference substrate, and the scanning test range was set to 200~800 nm. Surface photovoltage spectrum (SPV) was obtained by a self-assembly system consisting of a sample chamber, a lock-in amplifier with a light chopper, and a 300 W Xenon lamp as light source. The photoelectrochemical performance of the samples was recorded on AUT302N electrochemical workstation (Metrohm) with standard three-electrode cell. Among them, the electrodes of catalyst sample, standard Ag/AgCl, and platinum were defined as the electrode of working, reference, and counter, respectively. The electrolyte solution was sodium sulfate (Na_2_SO_4_) solution with the concentration of 0.2 M, and the light source was LED light. Photoluminescence spectra analysis was carried out on an Edinburgh Instruments FLS920 spectrofluorimeter. Electron Paramagnetic Resonance (EPR) spectra for hydroxyl radicals (sample, 4 mg; DMPO, 0.22 M; aqueous solution volume, 2.0 mL) and superoxide radicals (sample, 4 mg; DMPO, 0.22 M; methanol solution volume, 2.0 mL) were provided both in dark and visible light irradiation at 3186 G and 9056.895 MHz by an ER200-SRC electron spin resonance spectrometer (Bruker, Germany). The magnetic field strength, microwave strength, and scan width were set to 0.2 mT, 1 mW, and 250 mT, respectively. The sample to be tested was placed inside the NMR tube, and the test was performed in air at room temperature. All structural optimization and property calculations were performed using the CASTEP program package based on density functional theory (DFT) in the Materials Studio 2017 R2. The Perdew Burke Ernzerh (PBE) of generalized gradient approximation (GGA) was selected for exchange correlation of the interaction between electrons. The kinetic cut-off energy of 380 eV was set. The plane wave function was used as basis sets. The calculations of the electronic state and state density were performed based on the optimized crystal structure.

## Results and Discussion

**Fig. 1:**
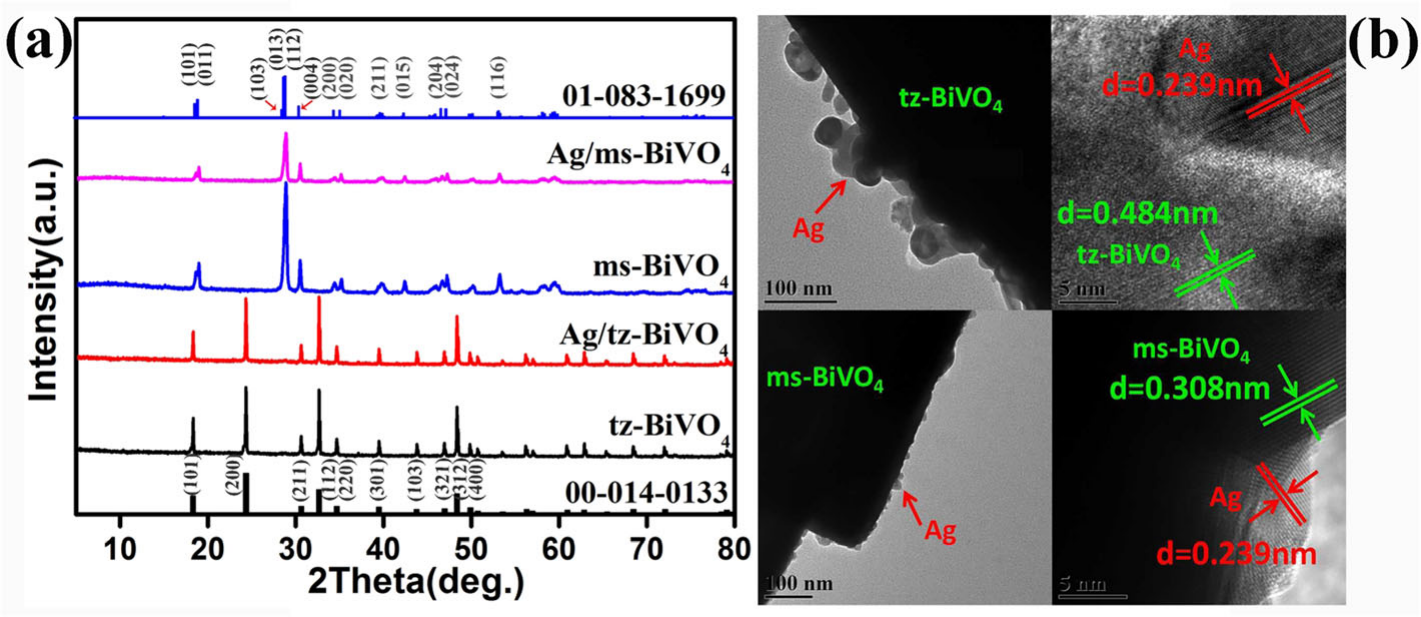
**a** XRD patterns of pristine tz-BiVO_4_, Ag/tz-BiVO_4_ sample, pristine ms-BiVO_4_, and Ag/ms-BiVO_4_ sample. **b** TEM image of Ag/tz-BiVO_4_, HRTEM of Ag/tz-BiVO_4_, TEM image of Ag/ms-BiVO_4_, and HRTEM of Ag/ms-BiVO_4_

XRD data imply that a phase structure variation from monoclinic scheelite (ms-) to tetragonal zircon (tz-) structure of BiVO_4_ can be achieved (Fig. S1). The junction of Ag nanoparticles led to no obvious alteration of the diffraction peaks of BiVO_4_ (Fig. 1a and Fig. S2). However, from Rietveld’s refined results, it is noted that either Ag/tz-BiVO_4_ or Ag/ms-BiVO_4_ showed an apparent lattice expansion in comparison to pristine tz-BiVO_4_ and ms-BiVO_4_ samples, which is summarized in Table S1. Lattice variation of BiVO_4_ matrix promised fine interfacial contact between Ag and BiVO_4_ nanoparticles, which is also proved by TEM observations. TEM and HRTEM images were given in Fig. 1b. Apparently, either ms-BiVO_4_ or tz-BiVO_4_ can act as a support to bind highly dispersed Ag nanoparticles, where the content of Ag nanoparticles is close to initial value as verified by EDS data (Fig. S3) [21, 22]. The d-spacing of 0.239 nm corresponds to the (111) plane of Ag (JCPDS No. 87-0597), whereas the adjacent lattice fringes of 0.308 nm and 0.484 nm are closely related to the (112) plane of ms-BiVO_4_ and (200) plane of tz-BiVO_4_, respectively.

**Fig. 2:**
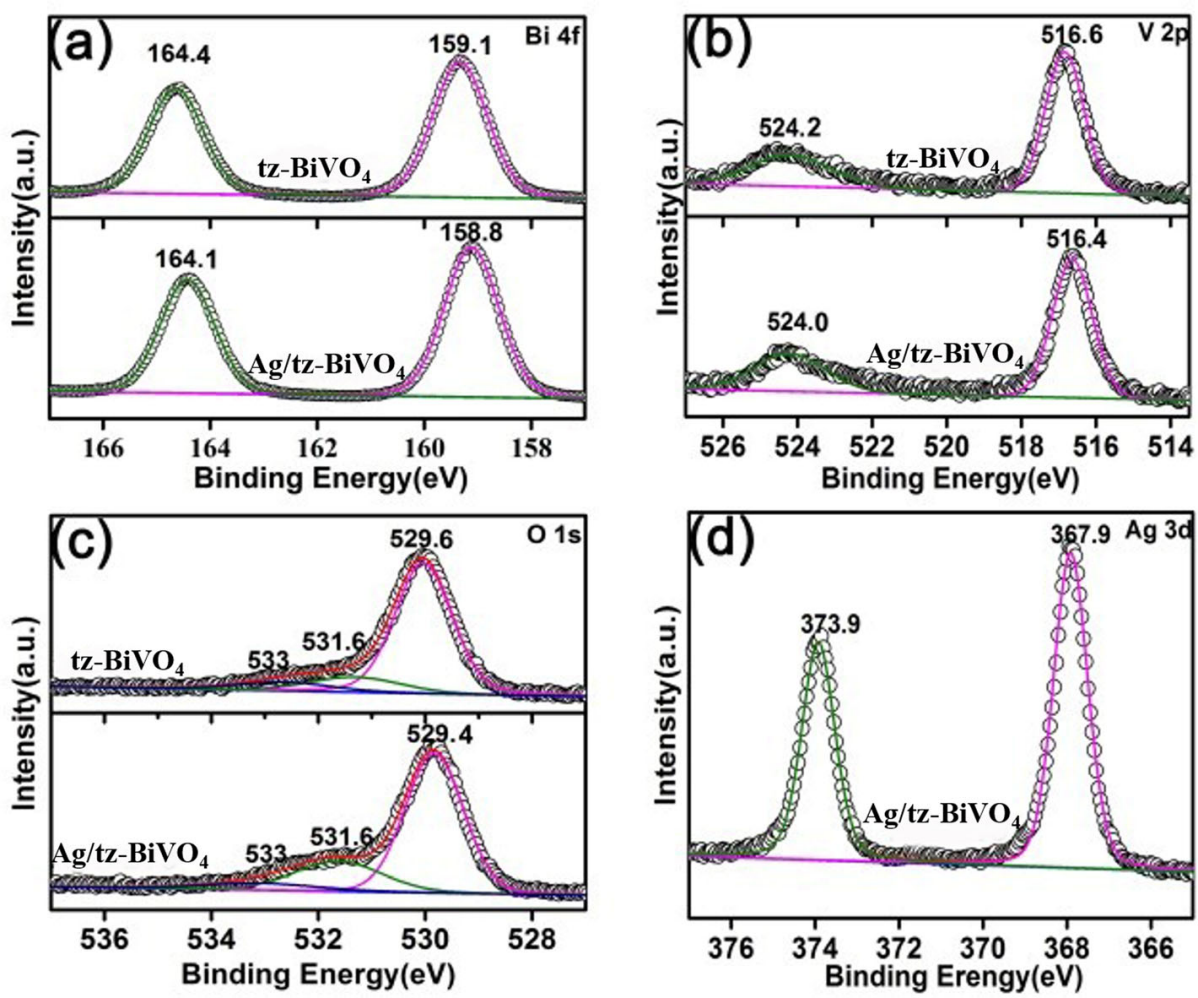
XPS spectra of tz-BiVO_4_ and Ag/tz-BiVO_4_ samples: (**a**) Bi 4f, (**b**) V 2p, (**c**) O 1 s, and (**d**) Ag 3d orbital

To acquire the surface chemical composition and oxidation states of the as-prepared samples, XPS technique was adopted. XPS results can fully verified that the Ag/BiVO_4_ catalyst was successfully prepared by the analyses of the binding energies over Bi, V, O, and Ag elements, as illustrated in Fig. 2 and Fig. S4. From Fig. 2a, it is seen that the Bi 4f orbital of tz-BiVO_4_ can be well-reproduced to two peaks with binding energies of 164.1 eV and 158.8 eV, which can be ascribed to the Bi 4f_5/2_ and Bi 4f_7/2_ orbitals, being close to the previous reported value [23, 24]. As for Ag/tz-BiVO_4_, a slight toward lower binding energy of about 0.3 eV was observed for Bi 4f orbital. Figure 2 b shows the high-resolution XPS data of V element. It is clearly that the binding energies of V 2p_1/2_ and V 2p_3/2_ locate at ~ 524.2 eV and 516.6 eV for pristine tz-BiVO_4_. Similar to that of Bi 4f orbital, V 2p orbital also gave a red shift of the binding energies for Ag/tz-BiVO_4_ heterojunction. Moreover, the O 1 s XPS analysis was also illustrated in Fig. 2c. Three typical binding energies of O 1 s orbital for tz-BiVO_4_ appears at 529.6 eV, 531.6 eV, and 533 eV, respectively, which can be assigned to the lattice oxygen, oxygen of surface hydration as well as the chemical-absorbed molecular O_2_, respectively [25]. As for Ag/tz-BiVO_4_, a red shift of ~ 0.2 eV was observed for the lattice oxygen in comparison to pristine tz-BiVO_4_. Such a behavior is supposed to be related to lattice expansion as well as the interfacial interaction between Ag and tz-BiVO_4_. Very often, the lattice expansion accompanies with elongated average lattice bonds and weakened strength of these bonds, which leads to the decrease of binding energies [26]. On the other hand, the variation of binding energies reflects the rearrangement of electron density near atoms, which can be influenced by surface modification. The decrease of the binding energies also implies the fine interfacial contact between Ag and tz-BiVO_4_, predicting an interfacial transfer may occur, which resulted in the variation of electron density [27]. This suspension can be verified by the following theoretical results. Moreover, XPS data also confirmed the metallic feature of Ag nanoparticles, and no evidence of Ag^+^ was observed in Ag/tz-BiVO_4_ heterojunction (Fig. 2d) [28]. On the other hand, the XPS results of Ag/ms-BiVO_4_ were also given in Fig. S4. Similar to that of Ag/tz-BiVO_4_ heterojunction, the binding energies of Bi 4f, V 2p, and O 1 s orbitals in Ag/ms-BiVO_4_ also exhibited a tiny redshift of about 0.1~0.2 eV. The slight variation of the binding energy shift in Ag/tz-BiVO_4_ and Ag/ms-BiVO_4_ is likely to be ascribed to the structural-dependent interfacial feature of BiVO_4_ and Ag nanoparticles.

**Fig. 3:**
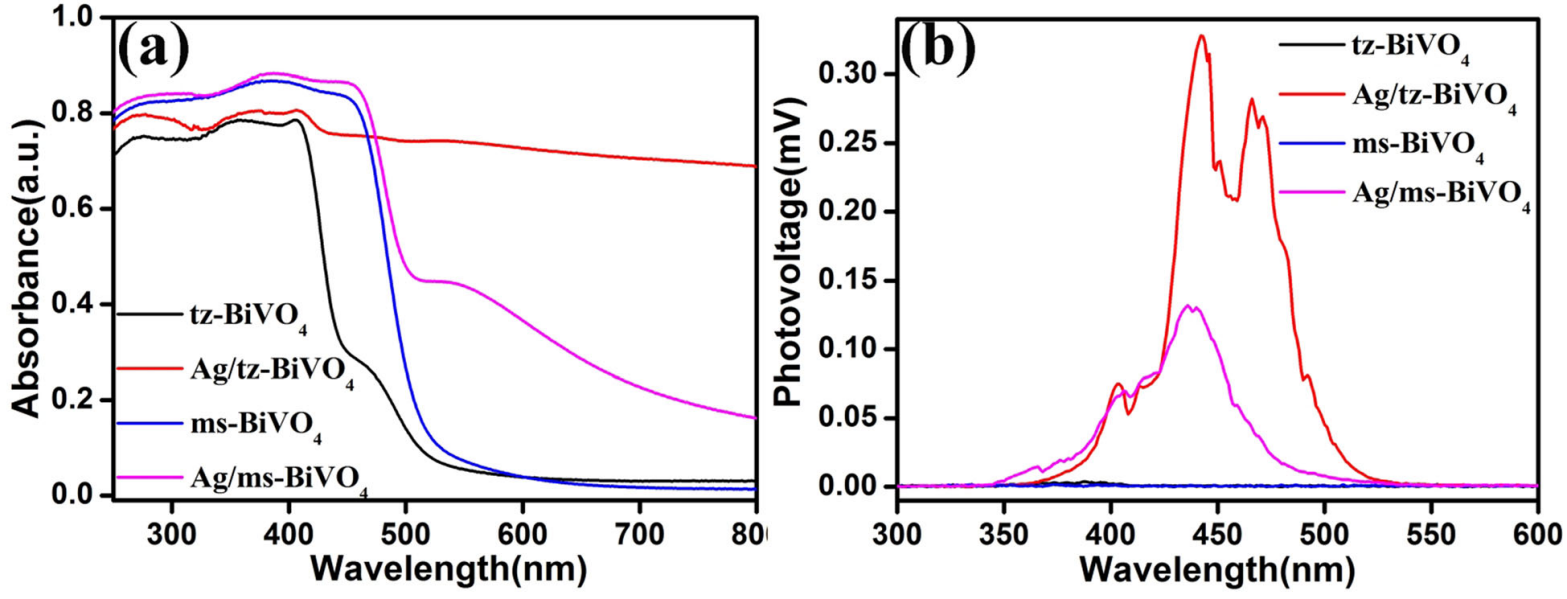
UV-visible diffuse reflectance spectra (**a**) and surface photovoltage spectra (**b**) of the as-prepared samples

Since the lattice expansion of BiVO_4_ occurred after Ag modification, the electronic structure may also be influenced, which can be verified by density functional theory (DFT) calculations. Because of large lattice mismatch between Ag and BiVO_4_, the convergence and structural optimization of Ag/BiVO_4_ is inaccessible. Thereby, a cluster/surface model was established to reveal the interfacial correlations between Ag and BiVO_4_ (Fig. S5 and Fig. S6). The band gap energy of tz-BiVO_4_ was estimated to be 2.59 eV, which is larger than that of 2.17 eV for ms-BiVO_4_ (Fig. S7), being in accordance to previous reported results [13, 29]. The anchoring of Ag cluster on BiVO_4_ surfaces has no obvious consequences on the typical electronic transitions from O 2p to V 3d orbital, as illustrated by the UV-visible diffuse reflectance spectra of the as-prepared samples (Fig. 3a). From Fig. 3a, it is seen that both ms-BiVO_4_ and tz-BiVO_4_ showed visible light response. According to Kubelka-Munk theory, the band gap energy of the samples can be calculated from the relationship between light absorption and band gap.

(*αhν*)^2^*= A*(*hν − E*_*g*_)

where *α*, *h*, *ν*, *E*_g_, and *A* mean absorption rate, Planck constant, frequency, band gap, and constant, respectively. The band gap energy of ms-BiVO_4_ and tz-BiVO_4_ was estimated to be 2.40 eV and 2.69 eV, respectively (Fig. S8), being close to the DFT results. It is noted the modification of Ag nanoparticles on BiVO_4_ surfaces led to an extension of the visible light absorption (Fig. S9). The broadened absorption should be originated to the SPR effects of Ag nanoparticles. Besides visible light absorption capability, the modification of Ag nanoparticle on BiVO_4_ can also has great impact on the kinetics of photoinduced charge carriers.

**Fig. 4:**
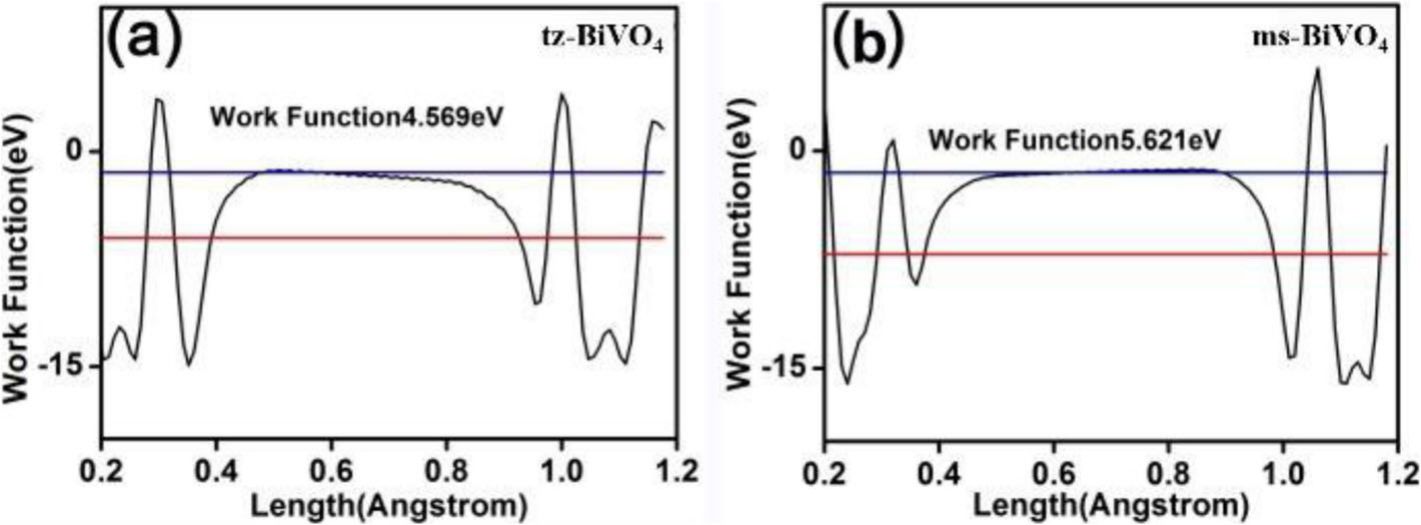
The work function of pristine tz-BiVO_4_ (**a**) and pristine ms-BiVO_4_ (**b**)

As illustrated by the surface photovoltage (SPV) signals in Fig. 3b, the maximal SPV signal of tz-BiVO_4_ was achieved to 0.33 mV after Ag modification, which is about 91.7 times higher than that of pristine tz-BiVO_4_. Moreover, it is noted that the intensity of SPV signal for Ag/tz-BiVO_4_ is also much higher than that of Ag/ms-BiVO_4_. Frequently, the SPV signal derived only from the photoinduced charge generation and separation process so that the intensity of the SPV signal reflects the efficiency of the charge carrier separation [30, 31]. Higher signals often predict improved charge separation efficiency, which suggests that stronger interaction between Ag and tz-BiVO_4_ exists than that for Ag and ms-BiVO_4_, being further clarified by DFT calculations. The atomic population analysis suggests that tz-BiVO_4_ acquired a net charge of about 0.33 e after Ag cluster modification. While for ms-BiVO_4_, only a small net charge of ~ 0.04 e occurs when anchored with Ag cluster (Table S2). Since the interfacial charge transfer highly depends on the Fermi levels as well as the electronic structures. To confirm the atomic population and the charge isolation between Ag and BiVO_4_, the work functions of BiVO_4_ and Ag/BiVO_4_ were given in Fig. 4. As illustrated in Fig. 4 a and b, the work functions for tz-BiVO_4_ and ms-BiVO_4_ were calculated to be 4.569 eV and 5.621 eV through aligning Fermi level to vacuum energy level (EVL). On the basis of the relationship between EVL and normal hydrogen electrode (NHE) [32], the Fermi levels of tz-BiVO_4_ and ms-BiVO_4_ were determined to be 0.069 V and 1.121 V, respectively. In view of solid-state physics, electrons can flow between hetero-interfaces, being highly dependent on the location of Fermi levels. Since the Fermi level of Ag locates at 0.4 V vs NHE, which is higher than that of tz-BiVO_4_ so that electrons will transfer from tz-BiVO_4_ surfaces to Ag. As a consequence, Ag is negatively charged, and tz-BiVO_4_ is positively charged, being in accordance to DFT results. This result predicts an internal electric field directed from Ag to tz-BiVO_4_, suggesting an efficient injection of photoinduced electrons from the conduction band of tz-BiVO_4_ to Ag would occur. As for ms-BiVO_4_, its lower Fermi level expects an inverse electron transfer process from Ag to ms-BiVO_4_. However, atomic population analysis demonstrated that no apparent electron migration between Ag and ms-BiVO_4_ was observed. This outcome may imply poor photoinduced charge carrier separation efficiency for Ag/ms-BiVO_4_ heterocatalyst.

**Fig. 5:**
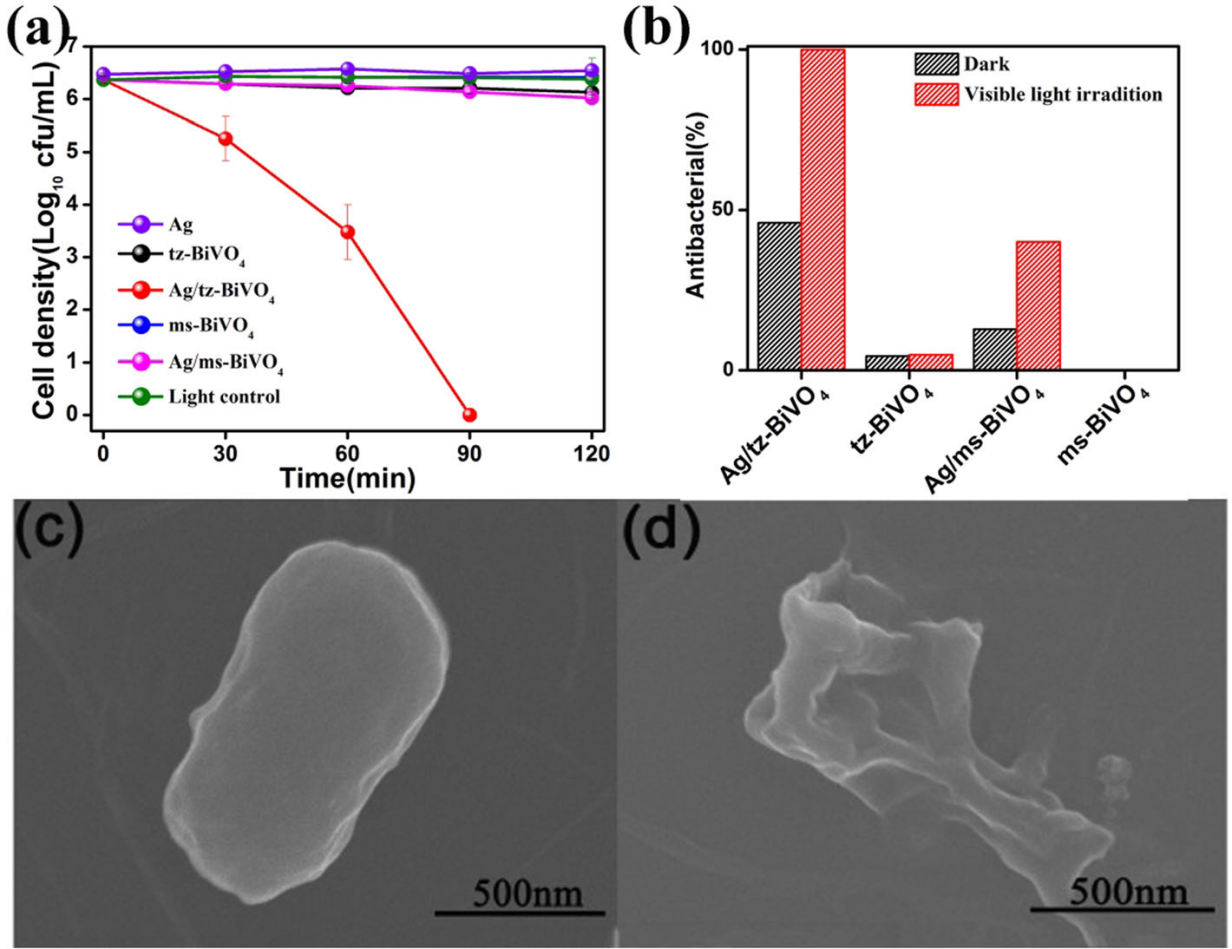
Photocatalytic inactivation of *E. coli* by Ag/tz-BiVO_4_ and Ag/ms-BiVO_4_ photocatalysts under VL irradiation (**a**). Comparison study of photocatalytic and thermocatalytic sterilization effect (**b**). SEM images of individual *E. coli* cell being photocatalytically inactivated by Ag/tz-BiVO_4_ for 0 h (**c**) and 2 h (**d**)

Having the above-mentioned results in mind, Ag/BiVO_4_ heterostructures would display structural-dependent photocatalytic performance. A wild bacterium, *E. coli*, was chosen as a model bacterium to study the photocatalytic inactivation activity of Ag/tz-BiVO_4_ and Ag/ms-BiVO_4_, respectively. Because *E. coli* is a Gram-negative bacterium, an auxiliary study was also carried out using a representative of Gram-positive bacteria with *S. aureus* (Fig. S10). A comparative study was firstly conducted to verify the inactivity of visible light toward the *E. coli* inactivation. As shown in Fig. 5a, the inactivation experiments of *E. coli* by tz-BiVO_4_, Ag/tz-BiVO_4_, ms-BiVO_4_, and Ag/ms-BiVO_4_ were carried out under visible light irradiation. It can be seen from Fig. 5a that the inactivation performance for *E. coli* over pristine tz-BiVO_4_ and ms-BiVO_4_ photocatalyst was merely detectable. However, anchoring of Ag nanoparticles can modulate the photocatalytic inactivation performance of BiVO_4_ (Fig. S11). Meanwhile, a structural-dependent photocatalytic performance was observed. When the weight ratio of Ag reached to 7%, Ag/tz-BiVO_4_ exhibited optimized photocatalytic inactivation efficiency over *E. coli* compared with some materials in the previous reports (Table S3). Within 90 min, the efficiency of bacterial inactivation reaches 100%, whereas Ag/ms-BiVO_4_ hetero-photocatalyst displayed tiny photocatalytic activity toward *E. coli* inactivation under VL irradiation (Fig. S11). As reported previously, Ag nanoparticles have found to show antibacterial activity. Thereby, a controlled experiment was carried in a dark chamber to testify the synergy of photocatalytic effect of Ag/BiVO_4_ heterostructures for the inactivation of *E. coli*. As shown in Fig. 5b, the inactivation process was conducted within 2 h under VL irradiation or in dark to compare photocatalytic and thermocatalytic sterilization effect of the as-prepared catalysts. It is found that ms-BiVO_4_ was inert to the inactivation of *E. coli*, while tz-BiVO_4_ exhibited poor activity either under VL irradiation or in dark. After Ag nanoparticles modification, the thermocatalytic activity was greatly improved. For instance, the thermocatalytic inactivation activity of Ag/tz-BiVO_4_ improved a factor of about 7.2 and 3.1 times higher than that of tz-BiVO_4_ and Ag/ms-BiVO_4_. Moreover, with VL irradiation, the catalytic activity of both Ag/tz-BiVO_4_ and Ag/ms-BiVO_4_ was predominately enhanced. The similar results can be obtained in the photocatalytic degradation of MB dye solution (Fig. S12a). After 7 h of visible light irradiation, the photocatalytic degradation rate of MB dye solution by 7Ag/tz-BiVO_4_ can reach about 85%. So as to know the destruction process of *E. coli* by Ag/tz-BiVO_4_, SEM observation was conducted to examine the morphology changes during the photocatalytic inactivation process as illustrated in Fig. 5 c and d. As shown in Fig. 5c, when the bacterial was not in contact with the catalyst, *E. coli* exhibited a well-preserved rod shape and intact cell structure. After 2 h irradiation reaction, disorganized membrane structures are observed (Fig. 5d), which demonstrates that the cell is completely decomposed. This matches well with the previous studies that photocatalytic treatment can induce significant disorder in membrane permeability of bacterial cells.

**Fig. 6:**
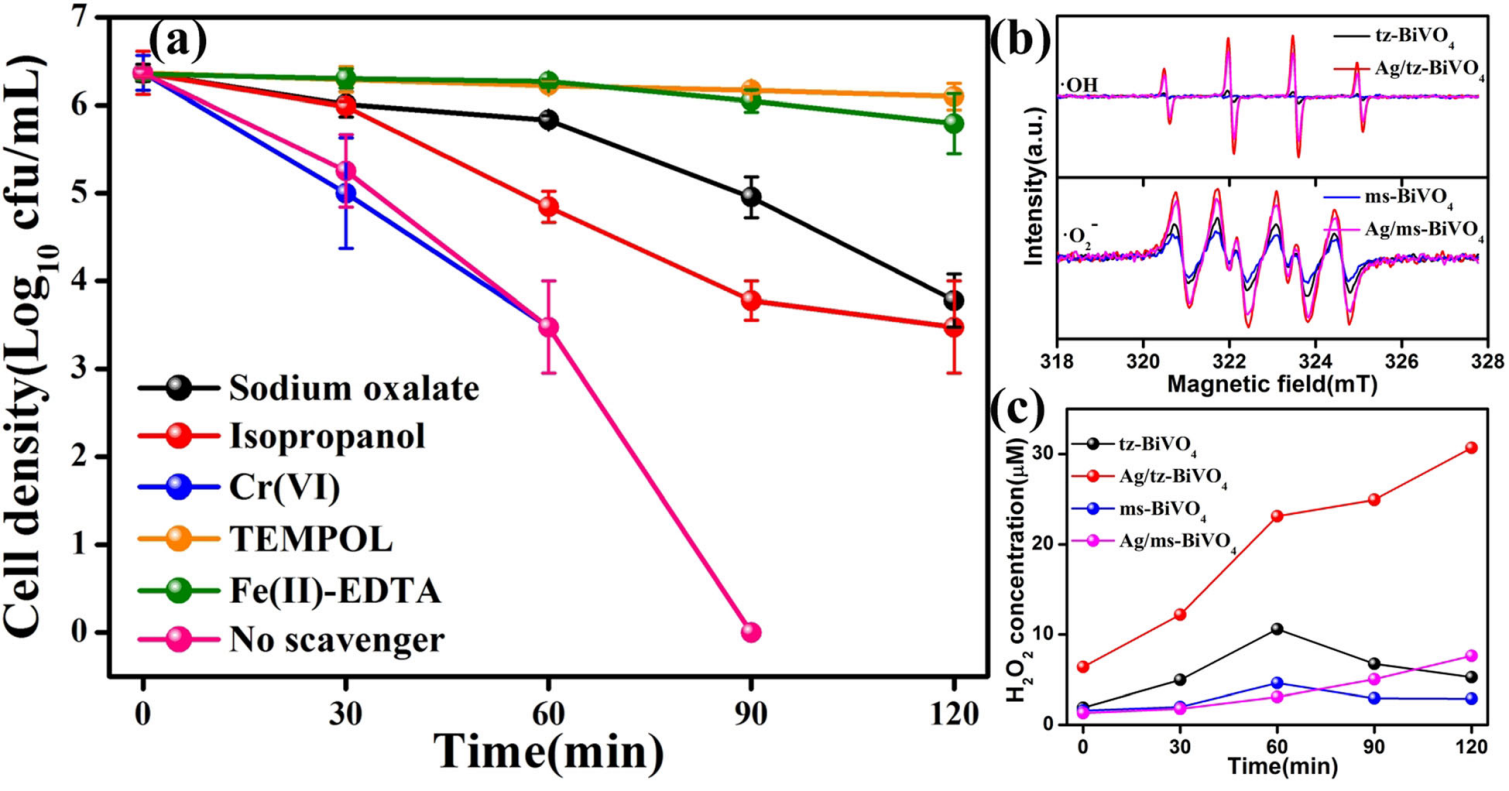
Photocatalytic inactivation efficiencies with respective scavengers in the presence of Ag/tz-BiVO_4_ (**a**). EPR spectra of •OH and DMPO-•O_2_^−^ in the presence of pristine tz-BiVO_4_, Ag/tz-BiVO_4_, pristine ms-BiVO_4_, and Ag/ms-BiVO_4_ under VL irradiation (**b**). Relative concentration of H_2_O_2_ by pristine tz-BiVO_4_, Ag/tz-BiVO_4_, pristine ms-BiVO_4_, and Ag/ms-BiVO_4_ (the pH of the suspension was adjusted to 9 using NaOH and took 3.5 mL; 50 μL of 0.7 mM lucigenin solution was added) (**c**)

To get further information of the photocatalytic process as well as the radical oxygen species that determine the inactivation process of *E. coli*, several types of radical species scavengers were carefully introduced by repeating the photocatalytic process of *E. coli* inactivation. As shown in Fig. 6a, sodium oxalate, isopropanol, Cr(VI), Fe(II)-EDTA, and Tetramethylpiperidine (TEMPOL) were taken as the scavengers for holes (h^+^), hydroxyl radicals (•OH), electrons (e^−^), H_2_O_2_, and superoxide radicals (•O_2_^−^) [33, 34]. Before the scavenger experiment was performed, the concentrations of different scavengers were optimized in earlier research. When no scavenger was added, 10^6^ cfu mL^−1^ of *E. coli* could be completely inactivated within 90 min. The bacterial inactivation is virtually suppressed with the addition of TEMPOL and Fe(II)-EDTA as the scavenger of •O_2_^−^ and H_2_O_2_, suggesting that •O_2_^−^ and H_2_O_2_ played critical roles in the photocatalytic inactivation process. After the addition of sodium oxalate and isopropanol, it can be observed that the bactericidal inactivation efficiency over Ag/tz-BiVO_4_ were partially inhibited, suggesting that h^+^ and •OH could directly destroy the *E. coli* cells with a powerful oxidation capability, whereas photoinduced electrons exhibited unobservable impact on the inactivation process of *E. coli*. And the capture experiment of photocatalytic degradation of MB dye solution was also carried out under visible light irradiation. In Fig. S12b, t-BuOH, silver nitrate (AgNO_3_), ethylenediaminetetraacetic acid (EDTA), and Fe(II)-EDTA were taken as the scavengers for •OH, e^−^, h^+^, and H_2_O_2_, respectively. The results indicate that H_2_O_2_ is the main active species in the experiment of photocatalytic degradation of MB dye solution. The active species •OH, e^−^, and h^+^ also have different effects on the photocatalytic degradation process, which is different from the role of active species in the photocatalytic sterilization capture experiment caused by errors in the plate counting method.

To acquire further information of the active species, electron paramagnetic resonance (EPR) measurement was used. In brief, DMPO acted as a spin trapper to testify the existence of •O_2_^−^ and •OH species [35, 36]. As displayed in Fig. 6b, very weak characteristic EPR signal of DMPO-•OH species was observed by prolonging VL irradiation time (Fig. S13). After Ag nanoparticles modification, the intensity of EPR signal of DMPO-•OH was drastically improved for both tz-BiVO_4_ and ms-BiVO_4_, suggesting that the capability to generate •OH for BiVO_4_ was greatly enhanced with Ag nanoparticles anchoring, being originated from the enhancement of charge carrier separation efficiency. Moreover, it is noted in Fig. 6c that the typical EPR signal of DMPO-•O_2_^−^ was also detected for all as-prepared samples (Fig. S14). Similar result to the EPR signal of DMPO-•OH is that the intensity of DMPO-•O_2_^−^ was also improved for Ag/tz-BiVO_4_ and Ag/ms-BiVO_4_ heterostructures. Interestingly, the EPR signal intensity of either DMPO-•OH or DMPO-•O_2_^−^ for Ag/tz-BiVO_4_ is higher than that for Ag/ms-BiVO_4_. For photocatalytic process, the electronic band potential always plays dominate roles in modulating the active species as well as the photocatalytic activity. On the basis of Mulliken electronegativity and band gap energy [37], the conduction band potentials of tz-BiVO_4_ and ms-BiVO_4_ were calculated to be about 0.21 V and 0.30 V versus NHE (S15). Thereby, the valence band potentials tz-BiVO_4_ and ms-BiVO_4_ were determined to be 2.90 V and 2.70 V versus NHE. According to previous literatures, the redox potential of •OH/H_2_O locate at 2.38 V versus NHE [38], suggesting the participation of •OH in the photocatalytic process for the inactivation of *E. coli*. However, it is seen that the redox potential of •O_2_^−^/O_2_ (− 0.33 V versus NHE) is more negative than the conduction potential of tz-BiVO_4_ and ms-BiVO_4_, indicating both tz-BiVO_4_ and ms-BiVO_4_ are not capable to generate •O_2_^−^ reactive species. This result seems to be contrary to the trapping experiments. Then, it is necessary to specify the origination of the •O_2_^−^ reactive species. In aqueous solution, a photoinduced hole can oxidize H_2_O_2_ to produce one •O_2_^−^ via the following equation: H_2_O_2_ + h^+^ → •O_2_^−^ + 2H^+^ [39]. Moreover, the generation •O_2_^−^ can also be achieved by reaction of H_2_O_2_ with •OH by the following equation: H_2_O_2_ + •OH → •O_2_^−^ + H_2_O + H^+^ [40]. From this point, the capability for the generation of H_2_O_2_ over the as-prepared BiVO_4_ samples should be investigated. The concentration of H_2_O_2_ as a function of VL irradiation time was given in Fig. 5c. Clearly, H_2_O_2_ can be generated for all as-prepared samples under VL irradiation. Predominantly, H_2_O_2_ concentration gradually increased from 6.40 to 30.69 μM in initial 120 min under VL irradiation for Ag/tz-BiVO_4_ heterostructure, which is much higher than the other samples. Consequently, junction of Ag and tz-BiVO_4_ can greatly improve the capability of the photocatalysts to generate H_2_O_2_ due to the fine interfacial contact, which resulted in highly improved photocatalytic activity toward *E. coli* inactivation as well as the phase dependent photocatalytic activity of Ag modified BiVO_4_ heterostructures.

As a result, a plausible explanation for the inactivation of *E. coli* over Ag/tz-BiVO_4_ was proposed. As the CB edge potential of tz-BiVO_4_ is higher than that of the metallic Ag nanoparticles, the electrons in the CB of tz-BiVO_4_ can quickly transfer toward to Ag nanoparticles, inhibiting the recombination of electron–hole pairs between the VB and CB of BiVO_4_. The photogenerated holes migrate to the surface of the semiconductor and then directly contact with bacteria, or even produce H_2_O_2_ and •OH with H_2_O molecules. Simultaneously, the enrichment of electrons on the Ag nanoparticles may be subsequently scavenged by H_2_O_2_ to produce •OH active species. The free radicals can react with the organic matter that constitutes the microbial organism and directly oxidize the organic matter into inorganic substances such as CO_2_ and H_2_O. This process will change the original state and properties of the microbial organism, thereby directly hindering the proliferation of microbial cells and preventing bacteria.

## Conclusions

In summary, Ag/BiVO_4_ heterostructural photocatalysts were developed with the aim to deliver a proof by rationally controlling the phase structure of BiVO_4_ and assembling Ag nanoparticles for photocatalytic antibacterial purpose in order to reveal structural-dependent photoinduced charge migration as well as the underlying photocatalytic antibacterial dynamic process. DFT theoretical calculation indicates an interfacial charge transfer between Ag and tz-BiVO_4_ with a net charge of about 0.33 e, which is far larger than that between Ag and ms-BiVO_4_, predicting fine interfacial contact and improved charge separation efficiency of Ag/tz-BiVO_4_. Relying on further experimental characterization, the optimized photocatalytic performance toward *E. coli* inactivation of Ag/tz-BiVO_4_ is predominately higher than that of tz-BiVO_4_, ms-BiVO_4_, and Ag/ms-BiVO_4_ catalysts. Besides photocatalytic activity, the thermocatalytic inactivation activity of Ag/tz-BiVO_4_ also exhibited a factor of about 7.2 and 3.1 times higher than that of tz-BiVO_4_ and Ag/ms-BiVO_4_. In combination with trapping experiment and EPR measurement, •O_2_^−^, •OH, and H_2_O_2_ active species played critical roles in the photocatalytic inactivation process. Moreover, detailed investigation suggested that the structural-dependent photocatalytic activity of Ag/BiVO_4_ mainly originated from the pronounced variation of the capability to produce H_2_O_2_ active species, where the capability of generating H_2_O_2_ over Ag/tz-BiVO_4_ is highly accelerated. This work provides hints for regulating the native properties of various structural-linked semiconductors.

## Supplementary information

**Additional file 1: Fig. S1.** XRD patterns of BiVO_4_ prepared at different pH value. **Fig. S2.** (a) XRD patterns of pure tz-BiVO_4_ and tz-BiVO_4_ samples loaded with various Ag content. (b)XRD patterns of pure ms-BiVO_4_ and ms-BiVO_4_ samples loaded with various Ag content. **Fig. S3.** Elemental composition profiles of Ag/tz-BiVO_4_. **Fig. S4.** XPS spectra of ms-BiVO_4_ and Ag/ms-BiVO_4_ samples: (a) Bi 4f, (b) V 2p, (c) O 1 s, (d) Ag 3d. **Fig. S5.** Crystal model of (a) tz-BiVO_4_, (b) ms-BiVO_4_. **Fig. S6.** Crystal model of (a) Ag/tz-BiVO_4_ (200), (b) Ag/ms-BiVO_4_ (121). **Fig. S7.** Band structure of (a) tz-BiVO_4_, (b) Ag/tz-BiVO_4_, (c) ms-BiVO_4_ and (d) Ag/ms-BiVO_4_. **Fig. S8.** Transformed Kubelka-Munk function ((αhν)^2^) versus light energy. **Fig. S9.** (a) UV-vis diffuse reflectance spectra of tz-BiVO_4_ and samples loaded with various wt% of Ag. (b) UV-vis diffuse reflectance spectra of ms-BiVO_4_ and samples loaded with various wt% of Ag. **Fig. S10.** Photocatalytic inactivation of E.coli and S.aureus by Ag/tz-BiVO_4_ photocatalysts under VL irradiation. **Fig. S11.** Photocatalytic inactivation of E.coli by tz-BiVO_4_ and samples loaded with various wt% of Ag under VL irradiation (a). Photocatalytic inactivation of E. coli by tz-BiVO_4_ and samples loaded with various wt% of Ag under VL irradiation (b). **Fig. S12** Photocatalytic degradation of MB dye solution by tz-BiVO_4_ and samples loaded with various wt% of Ag under visible light irradiation (a). Photocatalytic degradation of MB dye solution efficiencies with respective scavengers in the presence of Ag/tz-BiVO_4_ (b). **Fig. S13.** EPR spectra of •OH in the presence of tz-BiVO_4_ (a), Ag/tz-BiVO_4_ (b) ms-BiVO_4_ (c) and Ag/ms-BiVO_4_ (d) under dark and VL irradiation. **Fig. S14.** EPR spectra of DMPO-•O_2_^-^ in the presence of tz-BiVO_4_ (a), Ag/tz-BiVO_4_ (b) ms-BiVO_4_ (c) and Ag/ms-BiVO_4_ (d) under dark and VL irradiation. **S15.** The conducted band edge energy of a semiconductor. **Table S1.** Lattice parameters of the as-prepared samples. **Table S2.** Atomic Populations (Mulliken) of BiVO_4_ and Ag/BiVO_4_. **Table S3.** Comparison of Bactericidal Performance of photocatalysts.

## Data Availability

The datasets supporting the conclusions of this article are included within the article.

## Abbreviations

SEM: Scanning electron microscopy
TEM: Transmission electron microscopy
XRD: X-ray power diffraction
XPS: X-ray photoelectron spectroscopy
EPR: Electron paramagnetic resonance
SPV: Surface photovoltage spectrum
DFT: Density functional theory

## References

[1] Zou Y, Yang B, Liu Y, Ren Y, Ma J, Zhou X, Cheng X, Deng Y (2018). Controllable interface-induced co-assembly toward highly ordered mesoporous Pt@TiO_2_/g-C_3_N_4_ heterojunctions with enhanced photocatalytic performance. Adv. Funct. Mater.

[2] Yang C, Qin J, Xue Z, Ma M, Zhang X, Liu R (2017). Rational design of carbon-doped TiO_2_ modified g-C_3_N_4_ via in-situ heat treatment for drastically improved photocatalytic hydrogen with excellent photostability. Nano Energy.

[3] Haider M, Ijaz S, Ali J, Haider M, Imran H, Majeed I, Shahzadi MM, Ali JA, Khan MI (2020). Green synthesized phytochemically (Zingiber officinale and Allium sativum) reduced nickel oxide nanoparticles confirmed bactericidal and catalytic potential. Nanoscale Research Letters.

[4] Altaf S, Ajaz H, Imran M, Ul-Hamid A, Naz M, Aqeel M, Shahzadi A, Shahbaz A, Ikram M (2020) Synthesis and characterization of binary selenides of transition metals to investigate its photocatalytic, antimicrobial and anticancer efficacy. *Appl Nanosci*

[5] Aqeel M, Ikram M, Asghar A, Haider A, Ul-Hamid A, Naz M, Imran M, Ali S (2020). Synthesis of capped Cr-doped ZnS nanoparticles with improved bactericidal and catalytic properties to treat polluted water. Applied Nanoscience.

[6] Haider M, Ijaz M, Imran M, Naz H, Majeed JA, Khan MM, Ali MI (2020). Enhanced bactericidal action and dye degradation of spicy roots’ extract-incorporated fine-tuned metal oxide nanoparticles. Applied Nanoscience.

[7] Ikram M, Abbasi S, Haider A, Naz S, Hamid Anwar UI, Imran M, Ghaffar A (2020) Bi-metallic Ag/Cu incorporated into chemically exfoliated MoS_2_ nanosheets to enhance antibacterial potential: insilico molecular docking studies. *Nanotechnology*10.1088/1361-6528/ab8087 32182604

[8] Wahab M, Imran M, Ikram M, Naz M, Aqeel A, Rafiq H, Majeed SA (2019). Dye degradation property of cobalt and manganese doped iron oxide nanoparticles. Applied Nanoscience.

[9] Wang W, Li G, Xia D, An T, Zhao H, Wong PK (2017). Photocatalytic nanomaterials for solar-driven bacterial inactivation: recent progress and challenges. Environmental Science-Nano.

[10] McEvoy JG, Zhang Z (2014). Antimicrobial and photocatalytic disinfection mechanisms in silver-modified photocatalysts under dark and light conditions. Journal of Photochemistry and Photobiology C-Photochemistry Reviews.

[11] Park Y, McDonald KJ, Choi KS (2013). Progress in bismuth vanadate photoanodes for use in solar water oxidation. Chem. Soc. Rev.

[12] Ordon K, Kassiba A, Makowska-Janusik M (2016). Electronic, optical and vibrational features of BiVO_4_ nanostructures investigated by first-principles calculations. RSC. Adv.

[13] Dolic SD, Jovanovic DJ, Smits K, Babic B, Marinovic-Cincovic M, Porobic S, Dramicanin MD (2018). A comparative study of photocatalytically active nanocrystalline tetragonal zyrcon-type and monoclinic scheelite-type bismuth vanadate. Ceram. Int.

[14] Kweon KE, Hwang GS, Kim J, Kim S, Kim S (2015). Electron small polarons and their transport in bismuth vanadate: a first principles study. Phys. Chem. Chem. Phys.

[15] Zhang L, Ye X, Boloor M, Poletayev A, Melosh NA, Chueh WC (2016). Significantly enhanced photocurrent for water oxidation in monolithic Mo:BiVO_4_/SnO_2_/Si by thermally increasing the minority carrier diffusion length. Energy Environ. Sci.

[16] Guo K, Liu Z, Han J, Zhang X, Li Y, Hong T, Zhou C (2015). Higher-efficiency photoelectrochemical electrodes of titanium dioxide-based nanoarrays sensitized simultaneously with plasmonic silver nanoparticles and multiple metal sulfides photosensitizers. J. Power Sources.

[17] Chen D, Liu Z, Guo Z, Yan W, Xin Y (2018). Enhancing light harvesting and charge separation of Cu_2_O photocathodes with spatially separated noble-metal cocatalysts towards highly efficient water splitting. J. Mater. Chem. A.

[18] Kim JK, Shi X, Jeong MJ, Park J, Han HS, Kim SH, Guo Y, Heinz TF, Fan S, Lee CL, Park JH, Zheng XL (2018). Enhancing Mo:BiVO_4_ solar water splitting with patterned Au nanospheres by plasmon-induced energy transfer. *Adv*. Energy Mater.

[19] Ou M, Wan S, Zhong Q, Zhang S, Song Y, Guo L, Cai W, Xu Y (2018). Hierarchical Z-scheme photocatalyst of g-C_3_N_4_@Ag/BiVO_4_ (040) with enhanced visible-light-induced photocatalytic oxidation performance. *Appl. Catal*. B: Environ.

[20] Regmi C, Dhakal D, Lee SW (2018). Visible-light-induced Ag/BiVO_4_ semiconductor with enhanced photocatalytic and antibacterial performance. Nanotechnology.

[21] Xu X, Du M, Chen T, Xiong S, Wu T, Zhao D, Fan Z (2016). New insights into Ag-doped BiVO_4_ microspheres as visible light photocatalysts. RSC. Adv.

[22] Li M, Xu G, Guan Z, Wang Y, Yu H, Yu Y (2019). Synthesis of Ag/BiVO_4_/rGO composite with enhanced photocatalytic degradation of triclosan. *Sci*. Total Environ.

[23] Yang C, Li F, Li T (2015). A one-step ionic liquid-assisted ultrasonic method for the preparation of BiOCl/m-BiVO_4_ heterojunctions with enhanced visible light photocatalytic activity. CrystEngComm.

[24] Baral B, Reddy KH, Parida KM (2019). Construction of M-BiVO_4_/T-BiVO_4_ isotype heterojunction for enhanced photocatalytic degradation of norfloxacine and oxygen evolution reaction. J. Colloid Interf. Sci.

[25] Song M, Wu Y, Xu C, Wang X, Su Y (2019). Synergistic effects of multi-active sites in silver modified Bi°-BiVO_4_ toward efficient reduction of aromatic nitrobenzene. J. Hazard. Mater.

[26] Su Y, Zhu B, Guan K, Gao S, Lv L, Du C, Peng L, Hou L, Wang X (2012) Particle size and structural control of ZnWO_4_ nanocrystals via Sn^2+^ doping for tunable optical and visible photocatalytic properties. *J Phys. Chem* C 116:18508–18517

[27] Biesinger MC, Lau LWM, Gerson AT, Smart RSC (2012). The role of the auger parameter in XPS studies of nickel metal, halides and oxides. Phys. Chem. Chem. Phys.

[28] Xu M, Wang Y, Geng J, Jing D (2017). Photodecomposition of NO_x_ on Ag/TiO_2_ composite catalysts in a gas phase reactor. Chem. Eng. J.

[29] Safaei J, Ullah H, Mohamed NA, Noh MFM, Soh MF, Tahir AA, Ludin NA, Ibrahim MA, Isahak WNRW, Teridi MAM (2018). Enhanced photoelectrochemical performance of Z-scheme g-C_3_N_4_/BiVO_4_ photocatalyst. *Appl. Catal*. B: Environ.

[30] Somsongkul V, Lang F, Jeong AR, Rusu M, Arunchaiya M, Dittrich T (2014). Hole blocking PbI_2_/CH_3_NH_3_PbI_3_ interface. *Phys*. Status Solidi-R.

[31] Liu X, Liu Y, Gao F, Yang Z, Liu S (2016). Photoinduced surface voltage mapping study for large perovskite single crystals. Appl. Phys. Lett.

[32] Cheng J, Sprik M (2010). Aligning electronic energy levels at the TiO_2_/H_2_O interface. Phys. Rev. B.

[33] Wu D, Yue S, Wang W, An T, Li G, Yip HY, Zhao H, Wong PK (2016). Boron doped BiOBr nanosheets with enhanced photocatalytic inactivation of Escherichia coli. Appl. Catal. B: Environ.

[34] Xia D, Wang W, Yin R, Jiang Z, An T, Li G, Zhao H, Wong PK (2017). Enhanced photocatalytic inactivation of Escherichia coli by a novel Z-scheme g-C_3_N_4_/m-Bi_2_O_4_ hybrid photocatalyst under visible light: The role of reactive oxygen species. Appl. Catal. B: Environ.

[35] Khachatryan L, Vejerano E, Lomnicki S, Dellinger B (2011). Environmentally Persistent Free Radicals (EPFRs). 1. Generation of reactive oxygen species in aqueous solutions. Environ. Technol.

[36] Wang H, You C, Tan Z (2018). Enhanced photocatalytic oxidation of SO_2_ on TiO_2_ surface by Na_2_CO_3_ modification. Chem. Eng. J.

[37] Tang J, Zou Z, Ye J (2007). Efficient photocatalysis on BaBiO_3_ driven by visible light. J. Phy. Chem. C.

[38] Bard AJ, Parsons R, Jordan J (1985). Standard potentials in aqueous solution.

[39] Nosaka Y, Nosaka AY (2017). Generation and detection of reactive oxygen species in photocatalysis. Chem. Rev.

[40] Ishibashi K, Fujishima A, Watanabe T, Hashimoto K (2000). Quantum yields of active oxidative species formed on TiO_2_ photocatalyst. J. Photoch. Photobio. A.

