# Supplementary material for: Understanding the Structural-Dependent Photocatalytic Antibacterial Activity: a Case Study of Ag Modified BiVO4
Source: Nanoscale Res Lett. 2020 Jul 22;15:152. doi: 10.1186/s11671-020-03380-3 (PMC7376813; doi:10.1186/s11671-020-03380-3)
Supplement: Supplementary file 1 — Additional file 1: Fig. S1. XRD patterns of BiVO4 prepared at different pH value. Fig. S2. (a) XRD patterns of pure tz-BiVO4 and tz-BiVO4 samples loaded with various Ag content. (b)XRD patterns of pure ms-BiVO4 and ms-BiVO4 samples loaded with various Ag content. Fig. S3. Elemental composition profiles of Ag/tz-BiVO4. Fig. S4. XPS spectra of ms-BiVO4 and Ag/ms-BiVO4 samples: (a) Bi 4f, (b) V 2p, (c) O 1 s, (d) Ag 3d. Fig. S5. Crystal model of (a) tz-BiVO4, (b) ms-BiVO4. Fig. S6. Crystal model of (a) Ag/tz-BiVO4 (200), (b) Ag/ms-BiVO4 (121). Fig. S7. Band structure of (a) tz-BiVO4, (b) Ag/tz-BiVO4, (c) ms-BiVO4 and (d) Ag/ms-BiVO4. Fig. S8. Transformed Kubelka-Munk function ((αhν)2) versus light energy. Fig. S9. (a) UV-vis diffuse reflectance spectra of tz-BiVO4 and samples loaded with various wt% of Ag. (b) UV-vis diffuse reflectance spectra of ms-BiVO4 and samples loaded with various wt% of Ag. Fig. S10. Photocatalytic inactivation of E.coli and S.aureus by Ag/tz-BiVO4 photocatalysts under VL irradiation. Fig. S11. Photocatalytic inactivation of E.coli by tz-BiVO4 and samples loaded with various wt% of Ag under VL irradiation (a). Photocatalytic inactivation of E. coli by tz-BiVO4 and samples loaded with various wt% of Ag under VL irradiation (b). Fig. S12 Photocatalytic degradation of MB dye solution by tz-BiVO4 and samples loaded with various wt% of Ag under visible light irradiation (a). Photocatalytic degradation of MB dye solution efficiencies with respective scavengers in the presence of Ag/tz-BiVO4 (b). Fig. S13. EPR spectra of •OH in the presence of tz-BiVO4 (a), Ag/tz-BiVO4 (b) ms-BiVO4 (c) and Ag/ms-BiVO4 (d) under dark and VL irradiation. Fig. S14. EPR spectra of DMPO-•O2- in the presence of tz-BiVO4 (a), Ag/tz-BiVO4 (b) ms-BiVO4 (c) and Ag/ms-BiVO4 (d) under dark and VL irradiation. S15. The conducted band edge energy of a semiconductor. Table S1. Lattice parameters of the as-prepared samples. Table S2. Atomic Populations (Mulliken) of BiVO4 and Ag/BiVO [file 11671_2020_3380_MOESM1_ESM.docx]

Supplementary Information **for**

Understanding the structural-dependent photocatalytic antibacterial activity: a case study of Ag modified BiVO_4_

Hailin Guan, Yuefeng Tian, Alideertu Dong, and Yiguo Su*

*Inner Mongolia Key Laboratory of Chemistry and Physics of Rare Earth Materials, School of Chemistry and Chemical Engineering, Inner Mongolia University, Hohhot, Inner Mongolia 010021, PR China*

Corresponding author. Tel: +86-471-4344579. E-mail address: cesyg@imu.edu.cn (Y. Su).


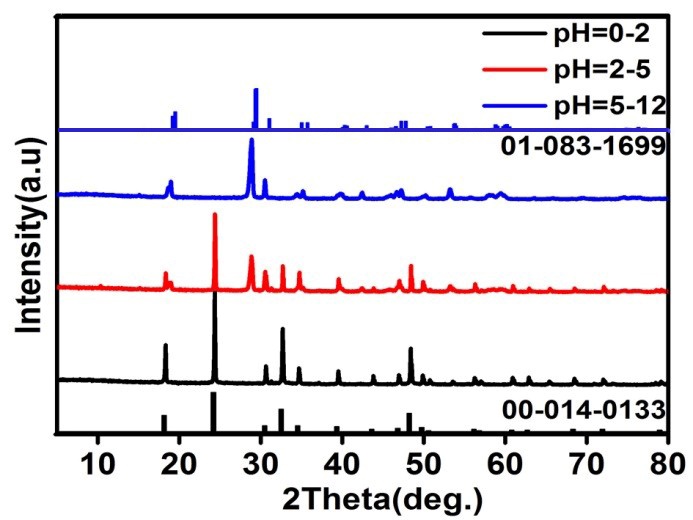


**Fig. S1** XRD patterns of BiVO_4_ prepared at different pH value.


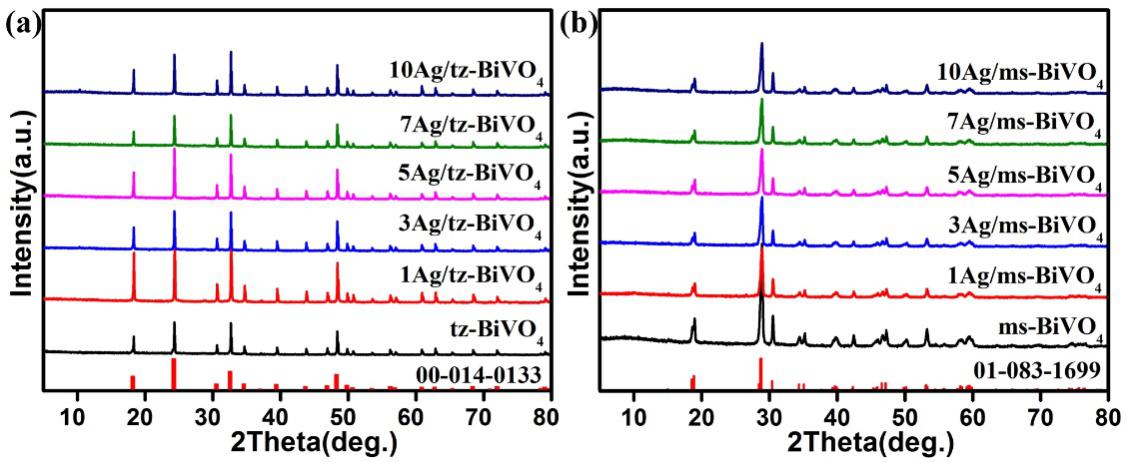


**Fig. S2** (a) XRD patterns of pure tz-BiVO_4_ and tz-BiVO_4_ samples loaded with various Ag content. (b)XRD patterns of pure ms-BiVO_4_ and ms-BiVO_4_ samples loaded with various Ag content.

**Table S1** Lattice parameters of the as-prepared samples.

| Sample | Lattice parameter | | |
| --- | --- | --- | --- |
|  | a (Ǻ) | b (Ǻ) | c (Ǻ) |
| tz-BiVO_4_ | 7.30727 | 7.30727 | 6.45688 |
| Ag/tz-BiVO_4_ | 7.31316 | 7.31316 | 6.45844 |
| ms-BiVO_4_ | 5.18996 | 5.10492 | 11.70639 |
| Ag/ms-BiVO_4_ | 5.22232 | 5.11338 | 11.70278 |


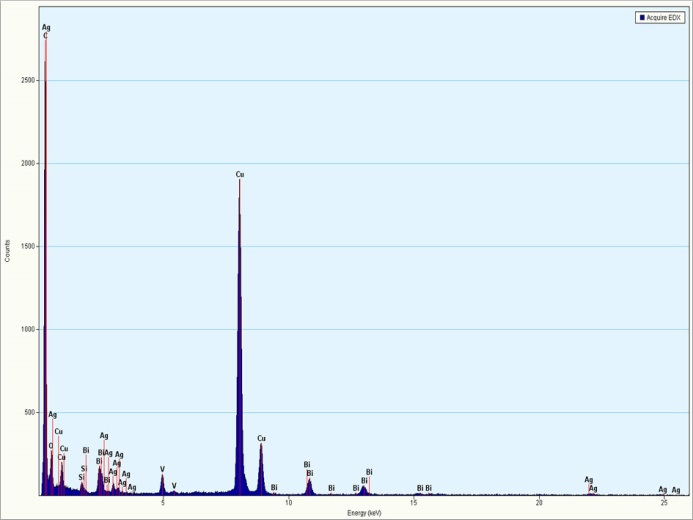


**Fig. S3** Elemental composition profiles of Ag/tz-BiVO_4_.


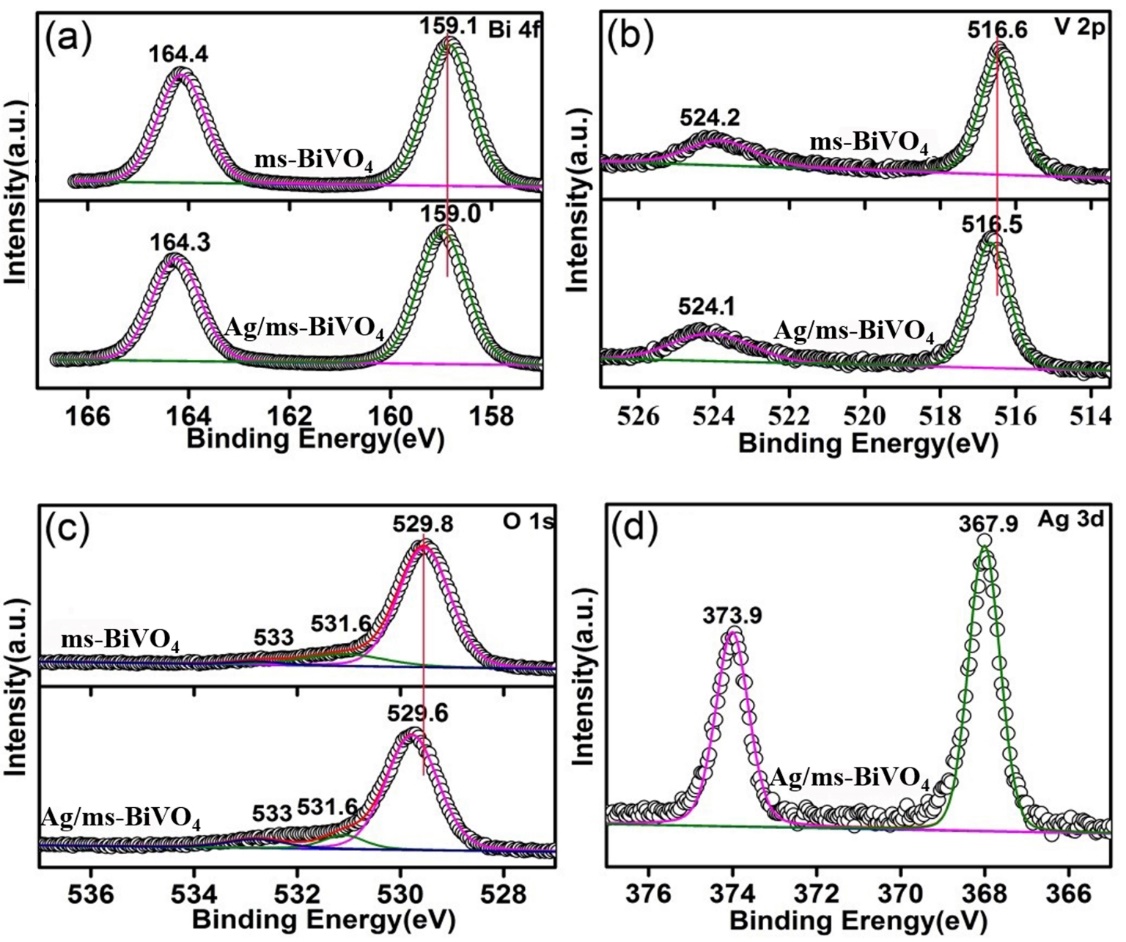


**Fig. S4** XPS spectra of ms-BiVO_4_ and Ag/ms-BiVO_4_ samples: (a) Bi 4f, (b) V 2p,

(c) O 1s, (d) Ag 3d.


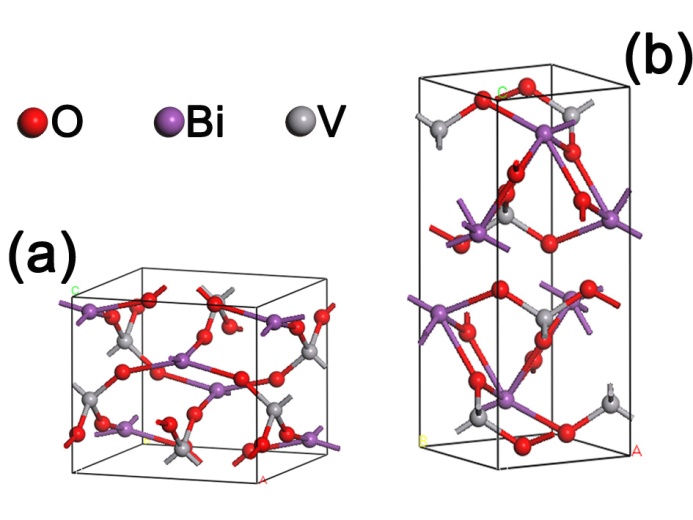


**Fig. S5** Crystal model of (a) tz-BiVO_4_, (b) ms-BiVO_4_.


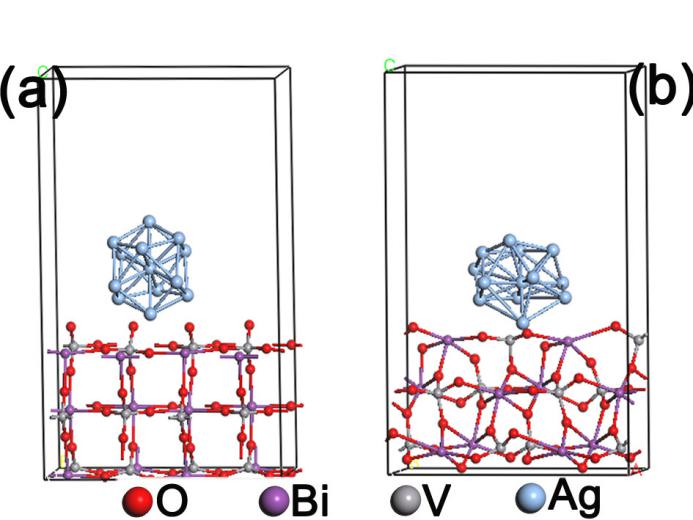


**Fig. S6** Crystal model of (a) Ag/tz-BiVO_4_ (200), (b) Ag/ms-BiVO_4_ (121).


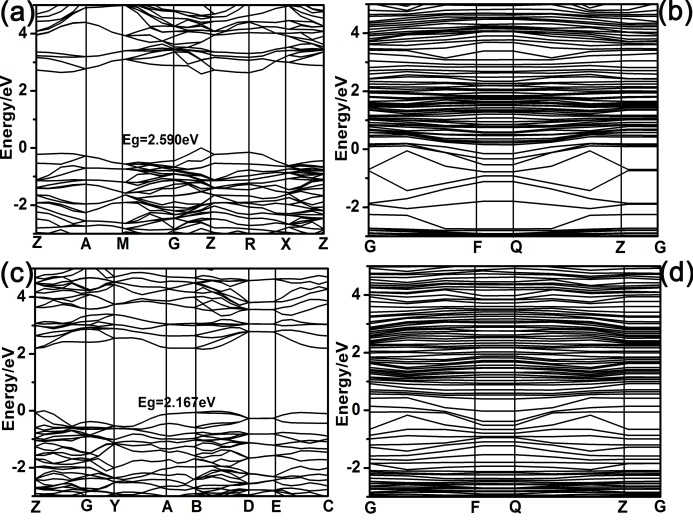


**Fig. S7** Band structure of (a) tz-BiVO_4_, (b) Ag/tz-BiVO_4_, (c) ms-BiVO_4_ and (d) Ag/ms-BiVO_4_.


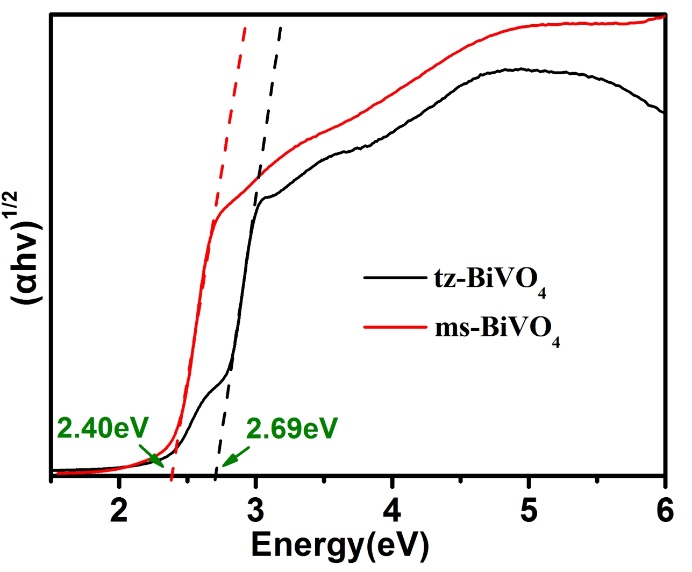


**Fig. S8** Transformed Kubelka–Munk function ((αhν)^2^) versus light energy.


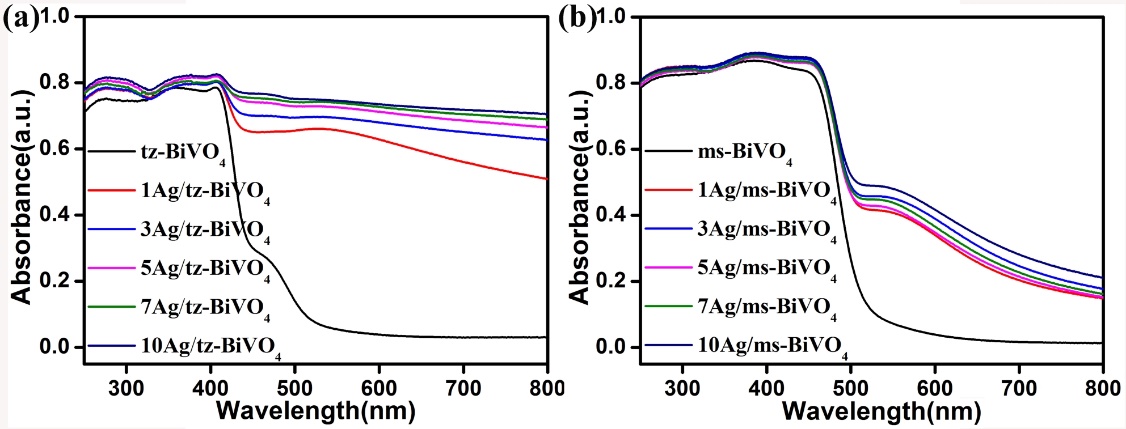


**Fig. S9** (a) UV–vis diffuse reflectance spectra of tz-BiVO_4_ and samples loaded with various wt% of Ag. (b) UV–vis diffuse reflectance spectra of ms-BiVO_4_ and samples loaded with various wt% of Ag.

**Table S2** Atomic Populations (Mulliken) of BiVO_4_ and Ag/BiVO_4_.

tz-BiVO_4_

| Atom | Orbits | Total Charge |
| --- | --- | --- |
|  | s p d f |  |
| O  V  Bi  Total | 1.88 4.82 0.00 0.00 6.70 -0.70  2.27 6.38 3.39 0.00 12.04 0.96  1.85 1.54 0.00 0.00 3.39 1.61  -0.01 | |

Ag/tz-BiVO_4_

| Atom | Orbits | Total Charge |
| --- | --- | --- |
|  | s p d f |  |
| O  V  Bi  Total | 1.88 4.81 0.00 0.00 6.69 -0.69  2.28 6.41 3.40 0.00 12.09 0.91  1.78 1.56 0.00 0.00 3.35 1.65  0.33 | |

ms-BiVO_4_

| Atom | Orbits | Total Charge |
| --- | --- | --- |
|  | s p d f |  |
| O  V  Bi  Total | 1.89 4.79 0.00 0.00 6.68 -0.68  2.19 6.35 3.37 0.00 11.91 1.09  1.89 1.48 0.00 0.00 3.38 1.62  0.02 | |

Ag/ms-BiVO_4_

| Atom | Orbits | Total Charge |
| --- | --- | --- |
|  | s p d f |  |
| O  V  Bi  Total | 1.89 4.79 0.00 0.00 6.68 -0.68  2.22 6.39 3.36 0.00 11.97 1.03  1.82 1.50 0.00 0.00 3.32 1.68  0.04 | |


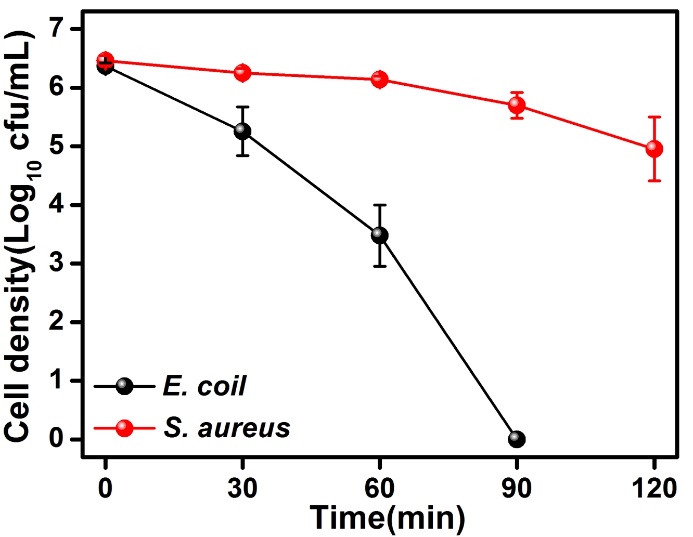


**Fig.** **S10** Photocatalytic inactivation of E.coli GB 8099 and S.aureus GB ATCC 6538 by Ag/tz-BiVO_4_ photocatalysts under VL irradiation.


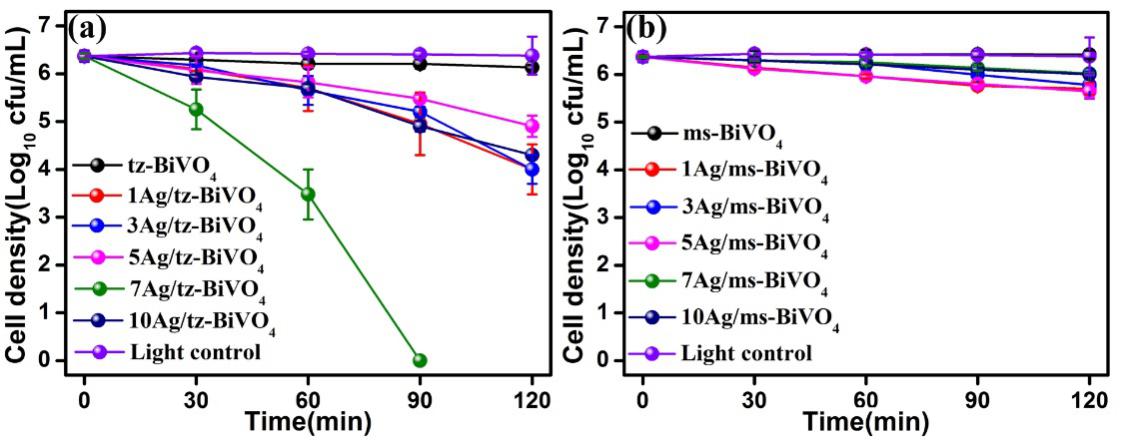


**Fig. S11** Photocatalytic inactivation of E.coli GB 8099 by tz-BiVO_4_ and samples loaded with various wt% of Ag under VL irradiation (a). Photocatalytic inactivation of E. coli GB 8099 by tz-BiVO_4_ and samples loaded with various wt% of Ag under VL irradiation (b).

**Table S3** Comparison of Bactericidal Performance of photocatalysts.

| Entry | Sample | Light source | | Time(min) | | Removal efficiency (%) | Ref. |
| --- | --- | --- | --- | --- | --- | --- | --- |
|  | Ag/BiVO_4_ | | Visible light | | 90 | 100 | This work |
|  | InVO_4_/BiVO_4_ | | 800W-Xenon lamp  _(420nm UV cutoff Filter)_ | | 300 | 99.89 | [1] |
|  | m-BiVO_4_ | | Visible light | | 120 | 96.5 | [2] |
|  | Ni/BiVO_4_ | | 150W short arc lamp  _(420nm UV cutoff Filter)_ | | 300 | 92 | [3] |
|  | Bi_2_S_3_/SnIn_4_S_8_ | | 300W Xe Lamp  _(420nm UV cutoff Filter)_ | | 300 | 100 | [4] |
|  | TiO_2_-Bi_2_WO_6_ | | 300W Xe Lamp  _(420nm UV cutoff Filter)_ | | 240 | 100 | [5] |
|  | GO/g-C_3_N_4_ | | 300 W xenon lamp  _(420nm UV cutoff Filter)_ | | 120 | 97.9 | [6] |
|  | Ag/PDA/g-C_3_N_4_ | | 300W Xe lamp  _(420nm UV cutoff filter)_ | | 120 | 99.2 | [7] |
|  | Ag-ZnO SRA | | 350W Xe lamp  _(420nm UV cutoff Filter)_ | | 150 | 99.9 | [8] |
|  | BiOI films | | 500 W Xe lamp  _(420nm UV cutoff Filter)_ | | 120 | 99.96 | [9] |
|  | PAN/Ag/TiO_2_ | | 250W metal halide lamp | | 120 | 95 | [10] |
|  | Cu-ZnO NPs | | Xe lamp  _(365nm UV cutoff Filter)_ | | 240 | 99 | [11] |
|  | Ag/ZnO/g-C_3_N_4_ | | 300 W Xenon lamp  _(420nm UV cutoff Filter)_ | | 120 | 100 | [12] |
|  | ZnO | | Pen-ray UV-C with light intensity of 5400µW/cm^2^ | | 210 | 99~100 | [13] |
|  | ZnO/TiO_2_ | | Visible light | | 90 | 92.7 | [14] |


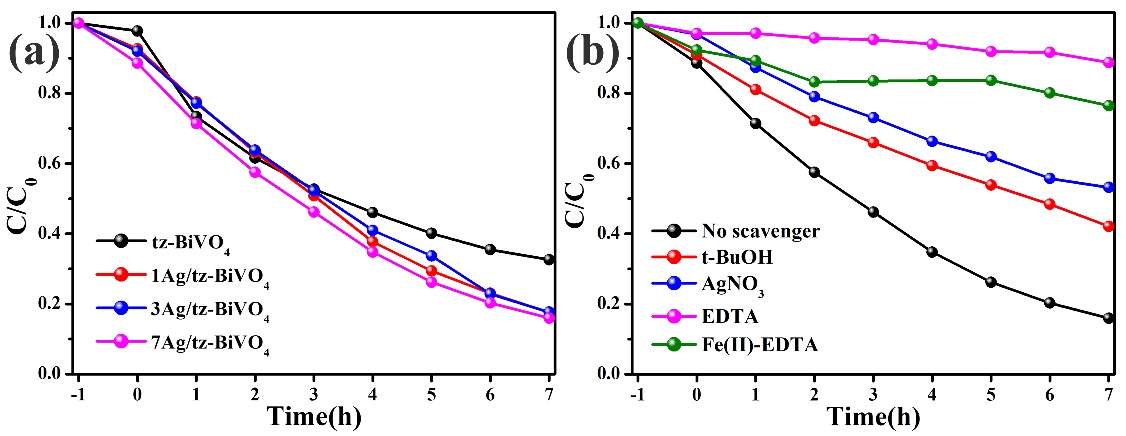


**Fig. S12** Photocatalytic degradation of MB dye solution by tz-BiVO_4_ and samples loaded with various wt% of Ag under visible light irradiation (a). Photocatalytic degradation of MB dye solution efficiencies with respective scavengers in the presence of Ag/tz-BiVO_4_ (b).


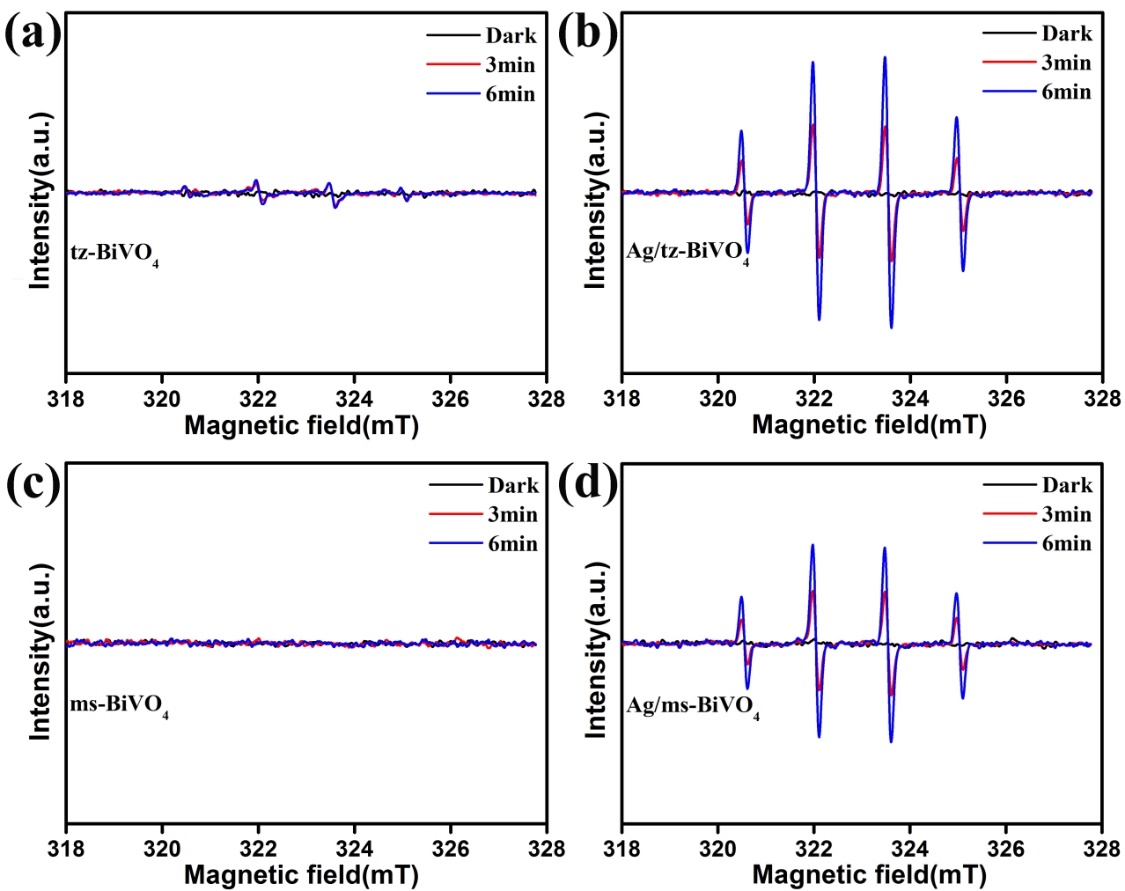


**Fig. S13** EPR spectra of •OH in the presence of tz-BiVO_4_ (a), Ag/tz-BiVO_4_ (b) ms-BiVO_4_ (c) and Ag/ms-BiVO_4_ (d) under dark and VL irradiation.


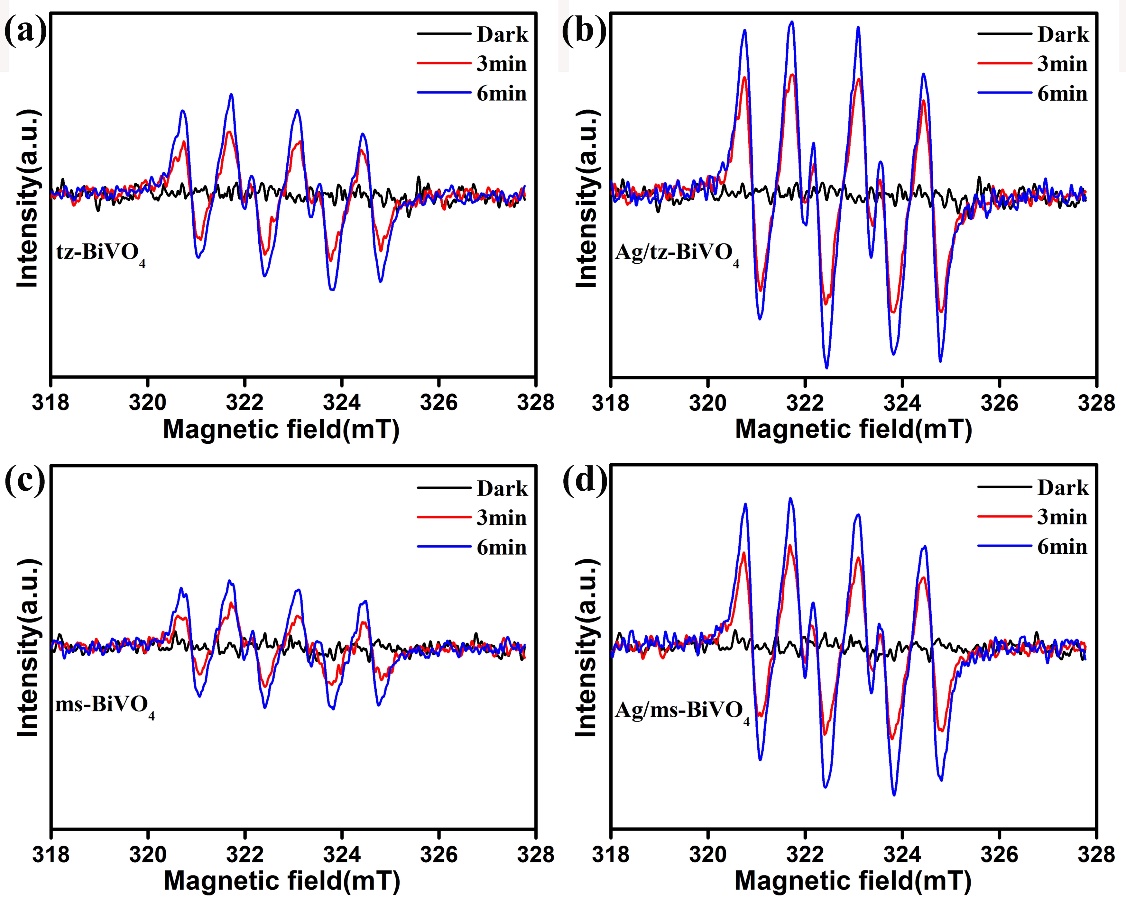


**Fig. S14** EPR spectra of DMPO-•O_2_^-^ in the presence of tz-BiVO_4_ (a), Ag/tz-BiVO_4_ (b) ms-BiVO_4_ (c) and Ag/ms-BiVO_4_ (d) under dark and VL irradiation.

**S15**

The conduction band edge energy of a semiconductor at the point of zero charge can be expressed by

*E_CB_* = *X* – *E^c^* – 0.5*E_g_*

where *E_CB_* is the conduction band edge potential and *X* is the Mulliken electronegativity of the semiconductor, which is the geometric mean of the electronegativities of the constituent atoms. *E^c^* is the energy of the free electron on the hydrogen scale, which is about 4.5 eV and *E_g_* is the band gap energy of BiVO_4_. The Mulliken electronegativity of an atom is the arithmetic mean of the atomic electron affinity and the first ionization energy. According to this expression, the rough conduction band potential was determined to be 0.21 V and 0.30 V with respect to normal hydrogen electrode for tz-BiVO_4_ and ms-BiVO_4_, respectively.

**References**

1. X. Zhang, J. Zhang, J. Yu, Y. Zhang, F. Yu, L. Jia, Y. Tan, Y. Zhu, B. Hou (2019) Enhancement in the photocatalytic antifouling efficiency over cherimoya-like InVO_4_/BiVO_4_ with a new vanadium source. *Journal of Colloid and Interface Science* 533:358-368
2. R. Sharma, Uma, S. Singh, A. Verma, M. Khanuja (2016) Visible light induced bactericidal and photocatalytic activity of hydrothermally synthesized BiVO_4_ nano-octahedrals. *Journal of Photochemistry and Photobiology B-Biology* 162:266-272
3. C. Regmi, Y.K. Kshetri, T.-H. Kim, R.P. Pandey, S.K. Ray, S.W. Lee (2019) Fabrication of Ni-doped BiVO_4_ semiconductors with enhanced visible-light photocatalytic performances for wastewater treatment. *Applied Surface Science* 475:1077-1077
4. H. Shi, Y. Zhao, J. Fan, Z. Tang (2019) Construction of novel Z-scheme flower-like Bi_2_S_3_/SnIn_4_S_8_ heterojunctions with enhanced visible light photodegradation and bactericidal activity. *Applied Surface Science* 465:212-222
5. Y. Jia, S. Zhan, S. Ma, Q. Zhou (2016) Fabrication of TiO_2_−Bi_2_WO_6_ Binanosheet for Enhanced Solar Photocatalytic Disinfection of E. coli: Insights on the Mechanism. *Acs Applied Materials & Interfaces* 8:6841-6851
6. L. Sung, T. Du, C. Hu, J. Chen, J. Lu, Z. Lu, H. Han (2017) Antibacterial Activity of Graphene Oxide/g‑C_3_N_4_ Composite through Photocatalytic Disinfection under Visible Light. *Acs Sustainable Chemistry & Engineering* 5:8693-8701
7. Y. Wu, Y. Zhou, H. Xu, Q. Liu, Y. Li, L. Zhang, H. Liu, Z. Tu, X. Cheng, J. Yang (2018) Highly Active, Superstable, and Biocompatible Ag/Polydopamine/g-C_3_N_4_ Bactericidal Photocatalyst: Synthesis, Characterization, and Mechanism. *Acs Sustainable Chemistry & Engineering* 6:14082-14094
8. J. Liu, J. Li, F. Wei, X. Zhao, Y. Su, X. Han (2019) Ag-ZnO Submicrometer Rod Arrays for High-Efficiency Photocatalytic Degradation of Congo Red and Disinfection. *Acs Sustainable Chemistry & Engineering* 7:11258-11266
9. Y. Wang, Y. Long, D. Zhang (2017) Facile in Situ Growth of High Strong BiOI Network Films on Metal Wire Meshes with Photocatalytic Activity. *Acs Sustainable Chemistry & Engineering* 5:2454-2462
10. L. Wang, J. Ali, C. Zhang, G. Mailhot, G. Pan (2020) Simultaneously enhanced photocatalytic and antibacterial activities of TiO_2_/Ag composite nanofibers for wastewater purification. *Journal of Environmental Chemical Engineering* 8
11. K. Qi, X. Xing, A. Zada, M. Li, Q. Wang, S.-y. Liu, H. Lin, G. Wang (2020) Transition metal doped ZnO nanoparticles with enhanced photocatalytic and antibacterial performances: Experimental and DFT studies. *Ceramics International* 46:1494-1502
12. S. Ma, S. Zhan, Y. Xia, P. Wang, Q. Hou, Q. Zhou (2019) Enhanced photocatalytic bactericidal performance and mechanism with novel Ag/ZnO/g-C_3_N_4_ composite under visible light. *Catalysis Today* 330:179-188
13. K.-A. Wong, S.-M. Lam, J.-C. Sin (2019) Wet chemically synthesized ZnO structures for photodegradation of pretreated palm oil mill effluent and antibacterial activity. *Ceramics International* 45:1868-1880
14. T. Nu Quynh Trang, L. Thi Ngoc Tu, S. Kim, D. Vinh Ai, J. Yi, V. Thi Hanh Thu (2020) High-Efficiency Photo-Generated Charges of ZnO/TiO_2_ Heterojunction Thin Films for Photocatalytic and Antibacterial Performance. *Journal of Nanoscience and Nanotechnology* 20:2214-2222
